# The mecillinam resistome in *Klebsiella pneumoniae*: how resistance to a one-target β-lactam triggers a diversity of responses

**DOI:** 10.1128/aac.01207-25

**Published:** 2026-02-03

**Authors:** Marie Royer, Nicolas Cabanel, Arnaud Gutierrez, Guilhem Royer, Olivier Barraud, Thierry Naas, Isabelle Rosinski-Chupin, Claude Loverdo, Philippe Glaser

**Affiliations:** 1 Ecology and Evolution of Antibiotic Resistance Unit, Institut Pasteur, CNRS UMR 6047 Université Paris Cité 555089, Paris, France; 2 Ecole doctorale complexité du vivant, Sorbonne Université 27063, Paris, France; 3 Université Paris Cité, CNRS, Inserm, Institut Cochin, Paris, France; 4 Université Limoges, INSERM, CHU Limoges, UMR 1092, Limoges, France; 5 Team "RESIST," Center for Immunology of Viral, Auto-immune, Hematological and Bacterial diseases (IMVA-HB/IDMIT/UMRS1184), Université Paris-Saclay, Inserm, CEA 546575 , Fontenay-aux-Roses, Le Kremlin-Bicêtre, France; 6 Laboratoire Jean Perrin, Sorbonne Université, CNRS 27063, Paris, France; 7 Institut de Biologie Paris-Seine, Sorbonne Université, CNRS, Inserm 27063, Paris, France; Columbia University Irving Medical Center, New York, New York, USA

**Keywords:** penicillin-binding protein, mecillinam, antibiotic resistance, heteroresistance, *Klebsiella pneumoniae*, β-lactam

## Abstract

Antimicrobial resistance is a silent pandemic responsible for 1.14 million deaths worldwide in 2021, with a major contribution from *Klebsiella pneumoniae*. β-lactams are the most commonly used antibiotics in humans, and there is an urgent need to characterize the resistance mechanisms to these antibiotics in Enterobacterales other than *Escherichia coli*. Mecillinam is a narrow-spectrum β-lactam targeting a single penicillin-binding protein, PBP2. Pivmecillinam, its oral prodrug, is used as a first-line treatment for uncomplicated urinary tract infections. It has been used for decades in Europe but was only authorized by the US Food and Drug Administration in 2024. Here, we decipher mecillinam resistance mechanisms in *K. pneumoniae* by characterizing its resistome in a pan-susceptible strain. The chromosomally encoded SHV β-lactamase led to spontaneous mecillinam-resistant mutants appearing at a higher rate, growing faster and at higher mecillinam concentrations in *K. pneumoniae* than in *E. coli*. The most frequent genetic event was an unstable duplication leading to heteroresistance. The selected mutations leading to resistance affected a wide range of functions, with resistance being dependent or independent of the RelA (p)ppGpp synthetase. Through an in-depth characterization of six mutant strains, we showed that, in the presence of mecillinam, they all experienced different growth defects despite high minimal inhibitory concentrations. Overall, our results in *K. pneumoniae* suggest different mechanisms to escape the complex mode of action of β-lactams in synergy with the β-lactamase SHV.

## INTRODUCTION

*Klebsiella pneumoniae* is one of the main contributors to the antimicrobial resistance burden. This species was responsible for an estimated 160,000 out of 1.14 million deaths attributable to antimicrobial resistance in 2021 (1) and for about a quarter of 462,500 deaths attributable to β-lactam resistance in 2019 (2). *K. pneumoniae* is a major cause of nosocomial infections, responsible mainly for septicemia, soft tissue, pulmonary, and urinary tract infections (UTIs) (3). It is highly permissive to horizontal gene acquisition and exhibits a high plasmid load capacity, which facilitates and contributes to the spread and evolution of antibiotic resistance (4). Almost all *K. pneumoniae* isolates carry a chromosomal *bla*_SHV_ gene, encoding an Ambler class A SHV β-lactamase (5, 6) that provides intrinsic ampicillin/amoxicillin resistance (7, 8).

β-lactams are the most prescribed antibiotics for humans because of their broad efficacy and their high tolerance (9). β-lactams target different penicillin-binding proteins (PBPs) depending on the molecule. Two PBPs are essential: PBP2, which is part of the rod system or elongasome, and PBP3 of the divisome (10). Following PBP inhibition by β-lactams, the bacterial cell wall loses its integrity, eventually leading to cell lysis. However, this effect does not explain the full complexity of the mode of action of β-lactams. Indeed, β-lactams trigger different metabolic and stress responses (11). It has been described in *Escherichia coli* that PBP2 or PBP3 transpeptidase activity inhibition leads to a futile cycle of peptidoglycan synthesis and turnover (12). The bacterium will recycle peptidoglycan precursors repeatedly until its lysis. Among β-lactams, the narrow-spectrum amidino-penicillin mecillinam (amdinocillin, MEC) specifically targets, in *E. coli* and *K. pneumoniae,* PBP2 (13, 14). Pivmecillinam, the oral prodrug of MEC, is used as a first-line treatment for community-acquired UTIs (15). It has been used for decades in Europe, particularly in Scandinavian countries (16), but was authorized in 2024 by the US Food and Drug Administration for uncomplicated UTIs (17).

The MEC resistome has been studied in *E. coli* as a model system to decipher the mode of action of β-lactam and analyze how the bacterium escapes β-lactam treatment (11, 12, 14, 18–20). Increased expression of the division protein FtsZ can bypass PBP2 inhibition (14). Different MEC^R^ mutants were reported or predicted to have an increased level of the divisome protein FtsZ compared to the wild-type (WT) strain (11, 21). *ftsZ* overexpression can be triggered by the stringent response (11, 14, 22). In *K. pneumoniae*, as in *E. coli*, the stringent response is mediated by the (p)ppGpp synthetase RelA and the bifunctional (p)ppGpp synthetase and hydrolase SpoT (23). Activation of the stringent response has been described in *E. coli* to protect bacteria from the lethal downstream effects of PBP inhibition (18, 24). Consistently, mutations in tRNA ligase genes (*argS*, *alaS*, *aspS, ileS, lysS, gltX,* and *thrS*) or in *spoT* are part of the *E. coli* global MEC resistome (18–21). In *E. coli*, mutations in amino acid metabolism genes, such as the L-cysteine biosynthesis regulator *cysB,* also conferred MEC resistance (19). By being auxotrophic for L-cysteine, the bacterium can activate an oxidative stress-like response to survive under MEC exposure. In *E. coli,* the MEC resistome also includes mutations in cell wall genes, such as the genes encoding the Tol-Pal system; in genes like *cydA* involved in the aerobic respiratory chain; or genes related to stress regulons like the Rcs phosphorelay and the general stress response mediated by RpoS (20, 21). However, despite the diversity of loci identified under laboratory conditions, mutation-based MEC resistance in clinical isolates mainly results from an alteration of the *cysB* gene (19).

Only a few clinical studies have analyzed the rate of MEC resistance in *K. pneumoniae*. A recent study analyzed UTI isolates from 2017 to 2020 in four different US regions (25). Among extended spectrum β-lactamase (ESBL)-negative isolates, 92.8% of *K. pneumoniae* isolates were susceptible to MEC compared to 97.5% of *E. coli*, whereas among ESBL-positive isolates, 80.0% of *K. pneumoniae* were susceptible compared to 98.2% of *E. coli*. Similar MEC resistance rates were observed in different studies analyzing *K. pneumoniae*-associated UTI ranging from 8.4% to 11.5% (26–29). This percentage increased between 17% and 91.5% in ESBL-producing isolates (29–36). Yet, in *K. pneumoniae*, the mechanisms of MEC resistance remain largely unknown. To our knowledge, there is no systematic study of the *K. pneumoniae* MEC resistome, and mutations that might contribute to MEC resistance in clinical isolates have not been reported.

Here, we decipher the mecillinam resistome of *K. pneumoniae* by selecting and sequencing genetic variants able to grow at high MEC concentrations. When compared to *E. coli*, MEC^R^ colonies appeared at a higher frequency, faster, and at higher MEC concentrations in *K. pneumoniae,* thanks to its chromosomally encoded SHV β-lactamase. MEC resistance resulted either from mutations in a broad range of functions reminiscent of those described in *E. coli* or from the unstable duplication of a chromosomal region encompassing the division and cell wall (*dcw*) locus, including *ftsZ*. In-depth characterization of selected MEC^R^ mutants from different pathways involved in MEC resistance revealed a diversity of growth defects in the presence of MEC in line with the complexity of MEC’s mode of action.

## RESULTS

### Spontaneous MEC^R^ colonies appear at higher MEC concentration and frequency in *K. pneumoniae* than in *E. coli*

**Fig 1:**
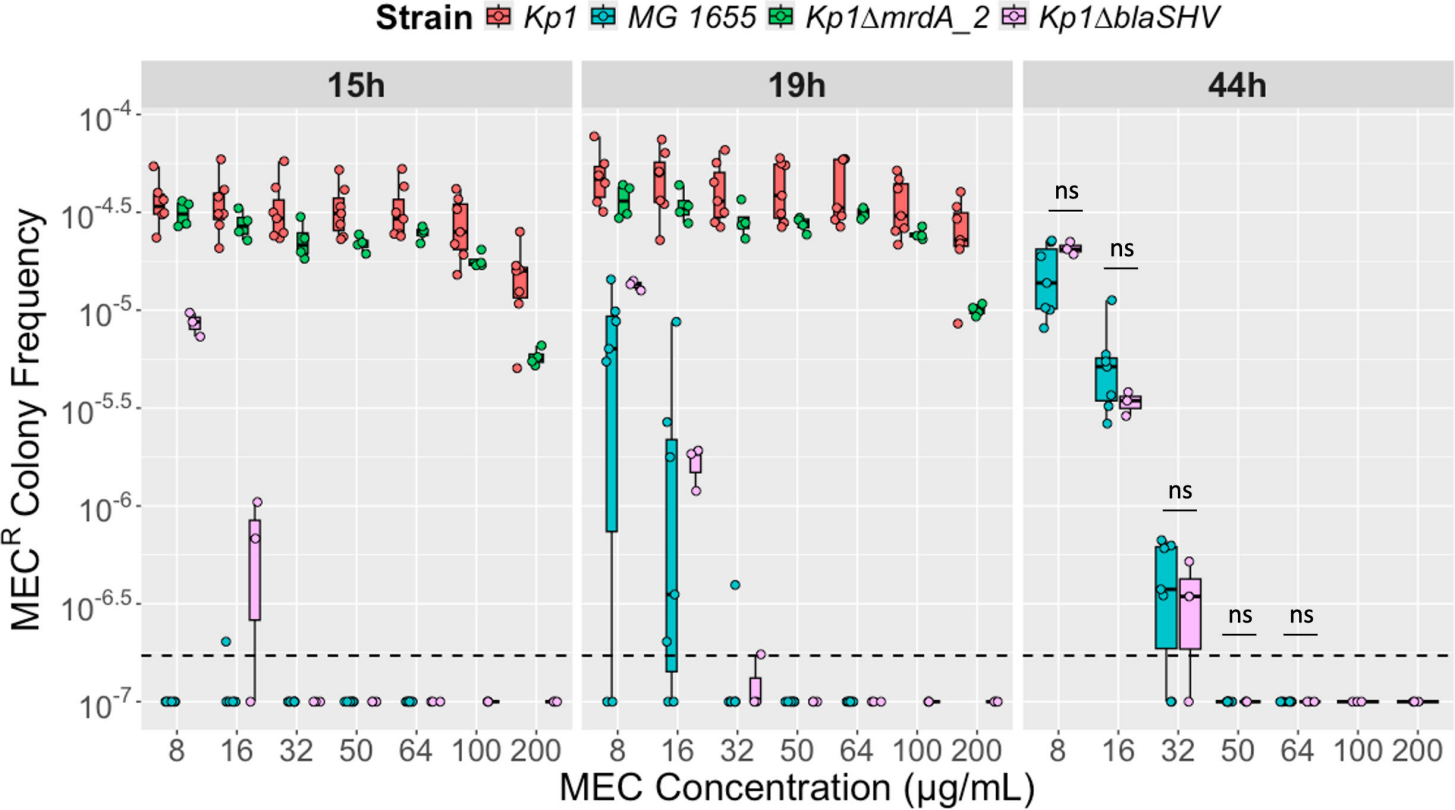
MEC^R^ colony frequencies of Kp1, its derivative strains, and *E. coli* MG1655. Diluted bacterial cultures were plated on MH agar plates supplemented with MEC at 8/16/32/50/64/100 and 200 µg/mL and incubated at 37°C. The MEC^R^ colony frequency for one strain is the ratio between CFUs per milliliter on MH+MEC and CFUs per milliliter on MH. MEC^R^ colony frequencies on MH+MEC of Kp1 (red), MG1655 (turquoise), Kp1∆*mrdA_2* (green), and Kp1∆*bla*_SHV_(pink). CFUs were counted at three incubation time points: 15 and 19 h for the four strains, and 44 h only for MG1655 and Kp1∆*bla*_SHV_. Experiments were performed with at least three replicates. Boxplots represent, from top to bottom, the third quartile, the median, and the first quartile. The dashed line corresponds to the lowest MEC^R^ colony frequency that can be measured (corresponding to one colony growing on MH+MEC plate). A three-way ANOVA (strain, time, and concentration) was performed, and as it was significant, we conducted multiple comparisons tests using Šidák or Tukey methods for *P*-value adjustments depending on the number of conditions compared. Here, we only show statistics of the comparison of the strains Kp1∆*bla*_SHV_ and MG1655 at the same MEC concentrations at 44 h. *P* values of the different comparisons are shown in Table S2. ns, non-significant.

In order to limit potential cross-resistance to other antibiotics, we selected, to characterize its MEC resistome, a pan-susceptible *K. pneumoniae* strain isolated from a rectal swab (Kp1; Table S1). First, we compared the frequency of MEC^R^ spontaneous mutants in Kp1 and in *E. coli* MG1655. Strains Kp1 and MG1655 showed similar MEC MICs of 0.22 and 0.25 µg/mL, respectively, as determined by E-test (Table S1). MEC^R^ mutants were selected by plating 100 µL at ≈6 × 10^7^ CFUs/mL of these two strains on MH agar plates supplemented with different MEC concentrations (8/16/32/50/64 µg/mL and with 100 and 200 µg/mL only for Kp1). We quantified MEC^R^ colony frequency for the different MEC concentrations after 15 and 19 h of incubation at 37°C for the two strains and after 44 h for *E. coli* only, as at that time point, Kp1 colonies became confluent (Fig. 1). We observed that (i) Kp1 MEC^R^ colonies were distinguishable earlier than for *E. coli*, as most of the Kp1 colonies had grown before 15 h compared to between 19 and 44 h for *E. coli*; (ii) the maximum MEC concentration tested allowing mutants' growth was higher, 200 µg/mL compared to 32 µg/mL for *E. coli* mutants (Fig. 1; Table S2). Furthermore, *E. coli* MEC^R^ frequency decreased with increasing MEC selective concentrations, but not for Kp1; and (iii) the frequency of Kp1 MEC^R^ colonies was higher, for example, 10 times more at MEC 8 µg/mL after 19 h (5.7 × 10^−5^ and 6.4 × 10^−6^ for Kp1 and MG1655, respectively) (Table S2C). Therefore, Kp1 genetic background predisposes this bacterium to become highly resistant to MEC (Fig. 1).

### The core genome encoded β-lactamase SHV allows MEC^R^ *K. pneumoniae* mutants to be selected at a higher MEC concentration than MEC^R^ *E. coli* mutants

**Fig 2:**
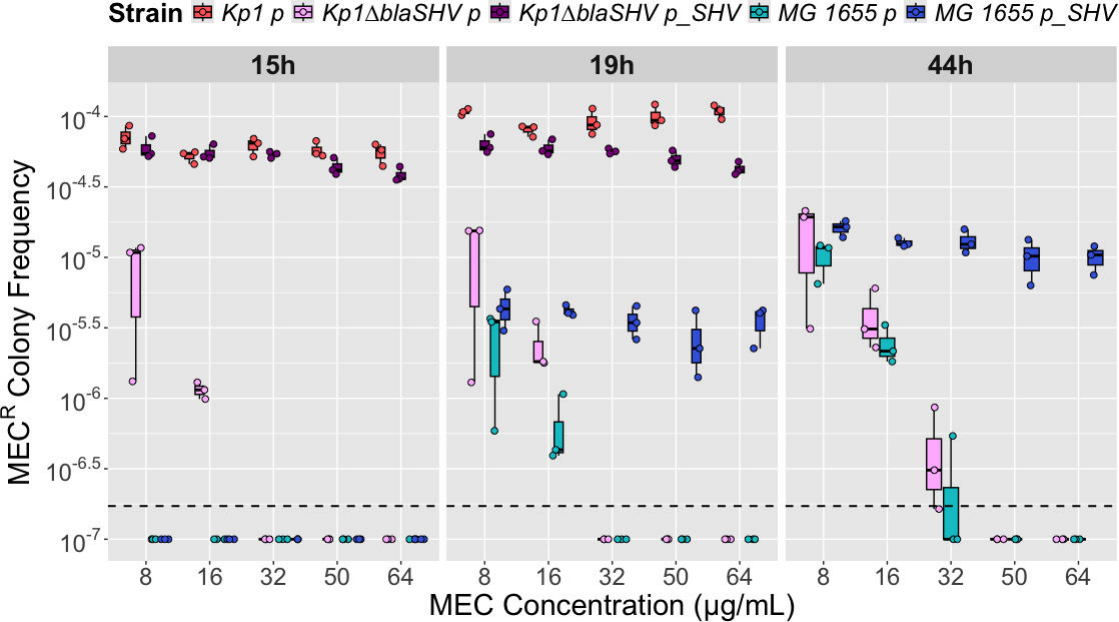
MEC^R^ colony frequencies following complementation by *bla*_SHV-11_ on the monocopy plasmid paGIBAC1. Diluted bacterial cultures were plated on MH agar plates supplemented with MEC at 8/16/32/50/64 µg/mL and incubated at 37°C. The MEC^R^ colony frequency for one strain is the ratio between CFUs per milliliter on MH+MEC and CFUs per milliliter on MH. MEC^R^ colony frequencies of Kp1 carrying the empty vector (Kp1 p) are in red, Kp1∆*bla*_SHV_ p is in pink, Kp1∆*bla*_SHV_ p_*bla*_SHV_ carrying the plasmid paGIBAC_*bla*_SHV_ is in purple, MG1655 p is in turquoise, and MG1655 p_*bla*_SHV_ is in dark blue. CFUs were counted at three incubation time points: 15, 19, and 44 h (except for Kp1 p and Kp1∆*bla*_SHV_ p_*bla*_SHV_). Experiments were performed in triplicate. Boxplots represent, from top to bottom, the third quartile, the median, and the first quartile. The dashed line corresponds to the lowest MEC^R^ colony frequency that can be measured (corresponding to one colony growing on MH+MEC plate). A three-way ANOVA (strain, time, and concentration) was performed, and as it was significant, we conducted multiple comparisons tests using the Šidák or Tukey methods for *P*-value adjustments depending on the number of conditions compared. The *P*-values of the different comparisons are shown in Table S3.

Compared to *E. coli*, *K. pneumoniae* has a gene paralogous to *mrdA* coding for the MEC target PBP2 (*mrdA_2*, 59% amino acid identity with *mrdA*). The biological function of this gene is unknown in *K. pneumoniae*. In addition, Kp1 expresses a core-genome β-lactamase, SHV-11, which is described as a non-ESBL penicillinase (8). We hypothesized that the product of one or both genes may enhance the ability of Kp1 to become resistant to higher MEC concentrations compared to *E. coli*. To test this hypothesis, we built two Kp1 derivative strains with deletions of these genes: Kp1∆*mrdA_2* and Kp1∆*bla*_SHV-11_, respectively. The two mutants have a MEC MIC similar to Kp1 of 0.22 µg/mL (Table S1). Next, we compared the frequency of MEC^R^ colonies on plates supplemented with different MEC concentrations. Kp1∆*mrdA_2* exhibited a modest reduction in the frequency of MEC^R^ colonies relative to Kp1, reaching statistical significance only at the highest MEC concentration (200 µg/mL) (Fig. 1; Table S2). For Kp1∆*bla*_SHV-11_, compared to Kp1, the frequency was reduced 4-fold at 8 µg/mL (1.4 × 10^−5^ vs 5.7 × 10^−5^) and 32-fold at 16 µg/mL (1.6 × 10^−6^ vs 5.2 × 10^−5^) at 19 h post-incubation. Moreover, Kp1∆*bla*_SHV-11_ MEC^R^ colonies grew only up to 32 µg/mL (Fig. 1). These frequencies were not different from those of *E. coli* at 44 h of incubation (Table S2C). We observed the same concentration-dependent decrease of MEC^R^ frequency as in *E. coli*, suggesting a role of the β-lactamase SHV-11 in the observed phenotype of Kp1. To sustain this hypothesis, we cloned *bla*_SHV-11_ under its own promoter in the monocopy vector paGIBAC1 (37) and introduced the resulting plasmid into Kp1∆*bla*_SHV-11_ and *E. coli* MG1655. The two transformants showed MEC MIC of 0.1575 and 0.125 µg/mL, respectively (Table S1). A similar analysis of the MEC^R^ colony frequency according to MEC concentration and time showed that plasmid expression of *bla*_SHV-11_ fully complemented the phenotype of Kp1∆*bla*_SHV-11_ (Fig. 2; Table S3). In *E. coli* MG1655, *bla*_SHV-11_ expression led to colony growth at higher MEC concentrations, like Kp1. The MEC^R^ colony frequency was constant from 8 to 64 µg/mL instead of decreasing with increasing MEC concentrations (Fig. 2; Table S3B). However, compared to Kp1 and the complemented Kp1∆*bla*_SHV-11_ strain, colonies grew only at 19 h onward and at a lower frequency (Fig. 2; Table S3). Therefore, the intrinsic β-lactamase SHV-11 influences the MIC reached by resistant colonies (more than 256 times the MIC of parental strains) in *E. coli* MG1655 and in Kp1. SHV-11 also increases the MEC^R^ colony frequency but has a stronger effect in Kp1. To test whether the higher MEC^R^ colony frequency in Kp1 compared to Kp1∆*bla*_SHV-11_ was due to an increased tolerance to MEC, we performed time-kill experiments. We tested 10 different MEC concentrations ranging from 1/4 the MIC (0.0625 µg/mL) to 64 times the MIC (32 µg/mL) (Fig. S1). We did not detect any difference between the two strains, showing that the higher MEC^R^ colony frequency was not due to a higher tolerance of the parental strain compared to the *bla*_SHV-11_-deficient strain.

To ascertain that the higher frequency and the growth at higher MEC concentrations were not specific to *K. pneumoniae* strain Kp1, we performed similar selection at 8 and 64 µg/mL MEC for seven additional clinical *K. pneumoniae* isolates and one ST131 *E. coli* strain (ARDIG-75 [38]; Table S1). The seven *K. pneumoniae* isolates carried different chromosomal *bla*_SHV_ genes (SHV-1, SHV-11, SHV-27, and SHV-119, with SHV-27 being an ESBL hydrolyzing cefotaxime [39]) and no acquired resistance gene, except strain S2D-LIM-KP146, which carried a tetracycline resistance gene, *tet(*D). The *E. coli* ST131 strain carried no acquired resistance gene. Like Kp1, we observed growth of colonies for the seven additional *K. pneumoniae* strains at similar frequencies on MH-MEC at 8 and 64 µg/mL after 18 h of incubation (Table S4). At this time point, no colony was visible for the two *E. coli* isolates, and after 44 h, they were only seen at the lowest MEC concentration. In addition, although variable and ranging from 1.7 × 10^−5^ to 9.8 × 10^−5^, the MEC^R^ colony frequency was always higher in *K. pneumoniae* than for *E. coli*. Therefore, the capacity of resistance to high MEC concentration and at high frequency is a general feature of *K. pneumoniae* and likely independent of the *bla*_SHV_ allele.

### Targeted genes in MEC^R^ mutants show a large diversity of functions

To identify functions targeted in the MEC^R^ variants, we Illumina-sequenced 87 randomly chosen colonies that grew at the highest MEC concentrations (50/100/200 µg/mL). We detected mutation events in 62 colonies. Overall, 90% were in coding regions, and 68% of colonies had a single mutation (Table S5). Candidate causative mutations of resistance were detected in a broad range of functional categories, including the cell envelope (25%), amino acid metabolism (14%), central carbon metabolism (16%), and translation (7%) (Table 1).

**TABLE 1:** Distribution of mutations in the 87 sequenced Kp1 MEC^R^ mutants

Category^*a*^	Gene	Function	Nbr^*b*^	Mutations^*c*^	Nb^*d*^
Cell wall (25%)	*cpxA*	Sensor protein, Cpx system	17	**(CTG)**_**4→3**_ **nt502**	16
				E91K	1
	*tolA*	Membrane protein, Tol/Pal system	3	Q100*	1
				∆1bp nt211	1
				(T)_5→4_ nt770	1
	*tolB*	Periplasmic protein, Tol/Pal system	1	**∆1bp nt867**	1
	*tolR*	Membrane protein, Tol/Pal system	1	∆1bp nt84	1
Amino acid metabolism (14%)	*cysB*	Transcriptional regulator, L-cysteine biosynthesis	6	IS903 nt885	4
				E136^*^	2
	*cysE*	Serine acetyltransferase, L-cysteine biosynthesis	1	∆54bp nt615	1
	*glnD*	Bifunctional uridylyltransferase/uridylyl removal	1	W661^*^	1
	*ntrC (glnG)*	Regulator, nitrogen regulation	1	**∆11bp nt1149**	1
	*ntrB (glnL)*	Sensor, nitrogen regulation	3	Q74^*^	3
Central carbon metabolism (16%)	*aceE*	Pyruvate dehydrogenase complex subunit E1	2	S646C	1
				Q553^*^	1
	*aceF*	Pyruvate dehydrogenase subunit E2	2	**∆1bp nt1234**	1
				Q291^*^	1
	ENJAKFCB_02678	Similar to malate:quinone oxidoreductase	1	T29N	1
	*ubiE*	Ubiquinone and menaquinone biosynthesis	1	G195D	1
	*ubiH*	Ubiquinone and menaquinone biosynthesis	3	(G)_9→8_ nt27	3
	ENJAKFCB_00729	Similar to thiamine pyrophosphate binding protein	1	I421S	1
	*ccmI*	C-type cytochrome biogenesis protein	1	IS903 nt400	1
	*ispA*	Geranyl transferase/farnesyl diphosphate synthase	1	∆42bp nt483	1
	*lipA*	Lipoyl synthase	2	G106E	1
				L217R	1
Translation (7%)	*epmB*	L-lysine 2,3 amino-mutase	1	+G nt115	1
	*efp*	Elongation factor P	1	IS903 nt157	1
	*glyQ*	Glycyl-tRNA synthetase subunit alpha	2	E205V	1
				N123K	1
	*spoT*	Bifunctional (p)ppGpp synthetase and hydrolase	2	(GC)_4→3_ nt1271	1
				**T162M**	1
Intergenic mutations (10%)	*ldcA/emtA*	*ldcA*: muramoyltetrapeptide carboxypeptidase *emtA*: membrane-bound lytic murein transglycosylase E	2	G→A nt −156	2
	*dauA/prs*	*dauA*: C4-dicarboxylic acid transporter; *prs*: ribose-phosphate pyrophosphokinase	5	C→A nt −120	5
	ENJAKFCB_05194/-	Hypothetical protein	1	C→T nt320	1
	*adk/htpG*	*adk*: adenylate kinase; *htpG*: chaperone protein	1	C→T nt −54	1
Others (32%)	No mutation		25		25
	ENJAKFCB_01858	Hypothetical protein	1	L229Q	1
	ENJAKFCB_03502	Hypothetical protein	1	∆12bp nt869	1
	ENJAKFCB_01775	Hypothetical protein	1	R656L	1

^*a*^ Percentages are calculated based on the total number of sequenced mutants, as some mutants harbored several mutations. The sum of percentages is higher than 100%.

^*b*^ Number of isolates mutated in the gene.

^*c*^ Asterisk means non-sense codon. In bold are mutations in the six strains selected for in-depth analysis.

^*d*^ Number of isolates with the same mutation.

Unexpectedly, in 25 strains (29%), we did not detect any mutation. In these clones, MEC susceptibility testing by disk diffusion showed no difference compared with Kp1. We hypothesized that this phenotypic instability, similar to heteroresistance (40), might result from an unstable resistance genotype lost during the subsequent steps of growth before sequencing and disk diffusion assay. To test this hypothesis, we performed a similar experiment of MEC^R^ mutant selection but added 40 µg/mL MEC during regrowth of clones to maintain the MEC selective pressure prior to DNA extraction. Out of the 14 sequenced MEC^R^ mutants (Tables S5 and S6), 6 harbored a large duplication of 802 kb (720 coding regions; Table S7) flanked by two ribosomal operons (Fig. S2). This unstable large duplication encompassed the *ftsZAQ* genes, coding for proteins of the divisome, which might be responsible for the MEC^R^ phenotype (Table S7).

To investigate whether the SHV-11 β-lactamase might affect paths to MEC resistance and therefore the panel of mutated functions, we Illumina-sequenced 37 Kp1∆*bla*_SHV-11_ MEC^R^ mutants selected on plates supplemented with MEC (8 or 16 µg/mL). Overall, 7 colonies showed the 802 kb amplification containing *ftsZAQ*, 30 colonies showed mutations in 21 different genes, and 2 colonies harbored both (Tables S5 and S8). The distribution of the mutated genes among the five functional categories (14% in cell wall, 27% in amino acid metabolism, 8% in central carbon metabolism, and 22% in translation) was similar to the one observed in Kp1. Therefore, the expression of SHV-11 affected the MEC^R^ colony frequency but likely not the targeted functional categories.

### MEC^R^ mutants are highly resistant to MEC but show diverse growth defects

To further characterize the diversity of responses to MEC and the underlying mechanism of resistance, we selected six Kp1 MEC^R^ mutants representing the four main functional categories: *cpxA* (CTG)_4→3_, *tolB* ∆1bp at nt 867, *cysB* E136*, *ntrC*(*glnG*) ∆11bp at nt 1,149, *aceF* ∆1bp at nt 1,234, and *spoT* T162M (Table 1; Tables S1 and S5). The *cpxA* gene was found mutated in 17 out of the 87 Kp1 mutants (16 with the same loss of a CTG leucine codon and 1 harboring a non-synonymous single nucleotide polymorphism [SNP] [E91K]), *tolB* in 1, *cysB* in 6 (1 with the same non-sense mutation and 4 with an IS insertion), *ntrC* in 1, *aceF* in 2, and *spoT* in 2 (Table 1; Table S5). CpxA is the sensor of the two-component system CpxAR involved in membrane stress (41), and TolB is part of the Tol-Pal system implicated in membrane invagination (42). CysB is the regulator of L-cysteine biosynthesis (43), and NtrC is the regulator of the two-component system NtrBC involved in nitrogen metabolism regulation (44). AceF is one subunit of pyruvate dehydrogenase, the first step enzyme of the tricarboxylic acid cycle (45). Finally, SpoT is the bifunctional (p)ppGpp synthetase and hydrolase involved in the stringent response (46). The T162M mutation is located in the SpoT hydrolase domain (46, 47). The selected *cpxA* and *aceF* mutants were isolated on MH+MEC 50 µg/mL, *tolB*, *ntrC,* and *cysB* at MH+MEC 100 µg/mL, and *spoT* at MH+MEC 200 µg/mL. However, for *cpxA* and *cysB,* MEC^R^ mutants in these genes have also been isolated at other MEC concentrations, and we did not find any evidence for a correlation between mutation identity and isolation at a specific MEC concentration (Table S5).

**Fig 3:**
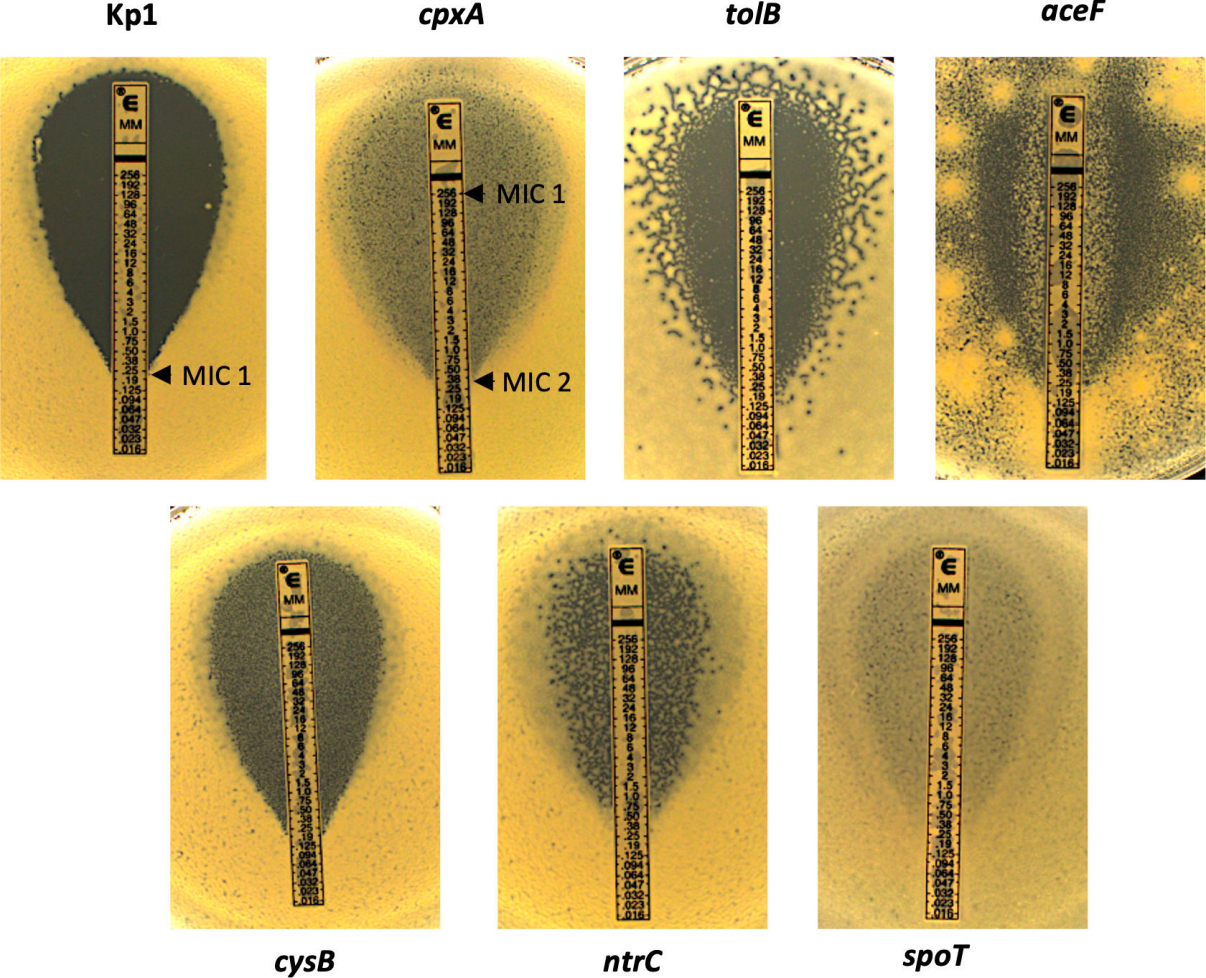
MEC MIC of Kp1 and *aceF, cpxA, ntrC, tolB, cysB,* and *spoT* MEC^R^ mutants. MIC (µg/mL) was measured by E-test after 16 h of incubation at 37°C with the flooding method. MIC_1 corresponds to the MIC measured according to EUCAST, and MIC_2 corresponds to the intersection between the thick and thin layers of bacteria (as shown for Kp1 and *cpxA*). Experiments were performed in triplicate. Median MIC values are given in Table S1.

The six mutant strains showed a MEC MIC beyond 256 µg/mL as determined by E-test (Fig. 3). These high MIC values were consistent with selection on plates at high MEC concentrations (50–200 µg/mL). However, we noticed a weaker layer of bacterial growth around the antibiotic strip forming a halo (Fig. 3). Hence, we determined a second MIC value (MIC_2) for each mutant, which corresponds to the boundary between the halo and the bacterial layer on the rest of the plate (Fig. 3; Table S1). MIC_2 was similar to Kp1 MIC (0.22 µg/mL), except for the *ntrC* and *cpxA* mutants, with MIC_2 of 1 and 0.38 µg/mL, respectively. To ascertain that the resistance phenotype was due to these mutations, we reintroduced the mutated alleles in the Kp1 strain (except for *aceF* and *spoT*, see also Methods). The MEC MICs of the engineered strains, like that of the original mutants, were above 256 µg/mL (Table S1; Fig. S3), demonstrating that the mutations were indeed responsible for MEC resistance.

In addition, the six mutants showed different growth patterns (Fig. 3). The *cpxA* and *spoT* mutants showed growth in the halo similar to that in the rest of the plate. The *cysB* mutant has a similar layer but thinner. The *ntrC* and *tolB* mutants showed heterogeneous growth, with some apparent lysis area more abundant for *tolB* and hardly visible growth in the halo. The *aceF* mutant grows better at the contact of the E-test, reminiscent of the Eagle effect (48). To evaluate the effect of the SHV β-lactamase on MEC resistance of the six mutants, we determined the MEC MIC in the presence of potassium clavulanate, an inhibitor of this β-lactamase. In all cases, we observed a strong decrease in the MIC, confirming the potentiator effect of the β-lactamase SHV-11 on MEC resistance (Table S9).

**Fig 4:**
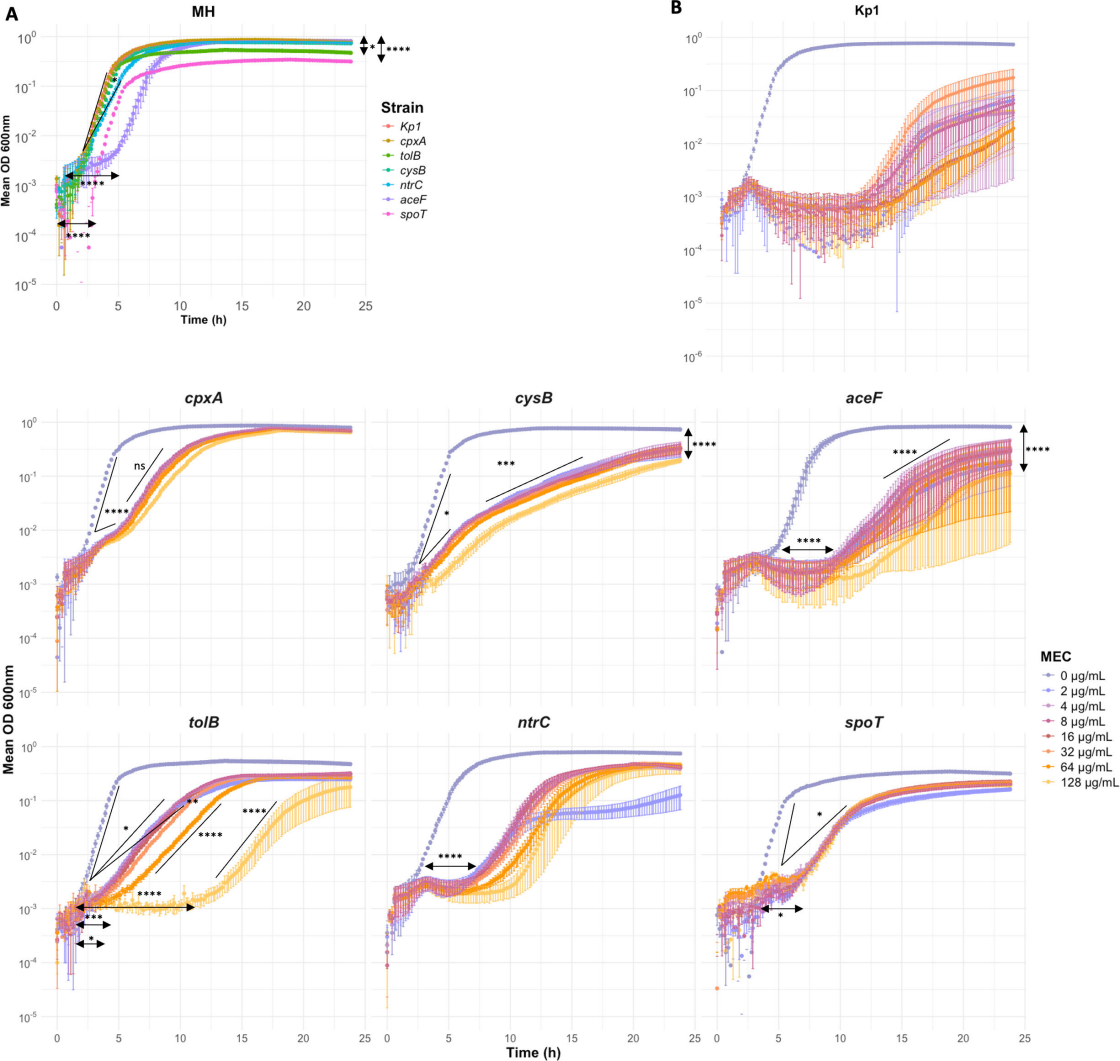
Bacterial growth of Kp1 and *aceF, cpxA, ntrC, tolB, cysB,* and *spoT* MEC^R^ mutants in MH broth supplemented with or without MEC. Bacterial cultures were diluted at an OD_600_ of 0.001 and dispensed in a 96-well plate with MH supplemented with or without MEC. The OD_600_ was measured for 24 h every 11 min. (**A**) Twenty-four hour growth curves of Kp1 (red), *aceF* (purple)*, cpxA* (brown)*, ntrC* (blue)*, tolB* (green)*, cysB* (turquoise), and *spoT* (pink) mutants in MH. (**B**) Growth curves of Kp1 and the six mutants with a gradient of MEC concentrations from 0 to 128 µg/mL according to the figure key. Each point represents the mean of at least three biological replicates, and the error bars represent the standard error. Growth curves are characterized according to three parameters: the lag time, the doubling time, and the maximal OD reached. The detailed values of these parameters are in Table S9. A two-way ANOVA (strain and concentrations) was performed to analyze these parameters. As the test was significant, we did multiple comparison tests with Šidák or Tukey methods for *P*-value adjustments depending on the number of conditions compared. In MH, we compared each growth parameter of the six mutant strains with Kp1 (**A**), and for each mutant strain and each growth parameter, we compared one MEC concentration with MH (**B**). *P < 0.05; **P < 0.01; ***P < 0.005; ****P < 0.001; and ns, non-significant. In some experiments, Kp1 can grow after 15 h in different MEC concentrations. We Illumina-sequenced the wells where we noticed growth for one experiment (MEC 2/4/8/64 µg/mL): three mutants were identified with the following mutations ∆36kb (34 hypothetical proteins), 30% of phage deletion, and *aspS* P179R for MEC 2, 4, and 64 µg/mL, respectively. We did not detect any mutation for MEC 8.

To further investigate bacterial growth in the inhibition halo, we analyzed the bacterial growth in MH broth for the six mutants compared to the parental strain and the impact of MEC. We monitored the OD_600_ of bacterial cultures supplemented with MEC for 24 h at concentrations ranging from 2 to 128 µg/mL. We analyzed growth curves according to three parameters: the lag time, the doubling time, and the maximum OD_600_ reached (Fig. 4; Table S10). Compared to the parental Kp1 strain, growth of four mutants was altered in the absence of MEC (Fig. 4A). The *aceF* and *spoT* mutants showed a longer lag time of +1.6 h, *ntrC* had a longer doubling time (+6.2 min), and *tolB* and *spoT* had a lower maximum OD_600_ reached (−0.3 and −0.5, respectively) (Table S10).

Next, we compared the three growth parameters for each mutant in the presence of MEC at different concentrations compared to their growth in the absence of MEC. The six mutants showed further growth defects in the presence of MEC, even though they all grew up to 128 µg/mL MEC (Fig. 4B). *ntrC*, *aceF*, *tolB,* and *spoT* mutants showed the strongest growth defects with longer lag time: +4.4, +6.7, +1.4, and +2.9 h at 16 µg/mL MEC, respectively (Table S10). Apart from *ntrC* and *aceF* under most MEC concentrations, all mutants displayed a significantly increased doubling time. The *cysB* and *aceF* mutants showed a lower maximum OD_600_ reached (−0.5 for both) (Table S10). According to a two-way (strain and concentration) ANOVA test, parameters were MEC concentration dependent for only two mutants. The *tolB* mutant showed an 8.1 h longer lag time between 64 and 128 µg/mL of MEC. The *ntrC* mutant showed a lower maximum OD_600_ reached at a MEC concentration of 2 µg/mL and a longer lag time at 128 µg/mL. Exponential growth curves showed two different slopes for the *cpxA*, *cysB,* and *aceF* mutants (Fig. 4B; Table S10). This might have resulted from changes in the composition of the MH medium during bacterial growth, affecting metabolism or stress pathways in these mutants. In the presence of MEC, as observed on plates, the growth of the six mutant strains in broth was altered, but in different ways.

In *E. coli*, an increase in the expression of *ftsZ* leads to resistance to A22, a drug inhibiting MreB, an essential component of the elongasome (12). We tested the growth of the six Kp1 MEC^R^ mutants studied earlier and the MEC^R^ strain MR271 harboring the 802 kb genomic duplication, including the *dcw* locus, on plates containing 10 µg/mL of A22. This A22 concentration inhibited the growth of Kp1, whereas the growth of the seven MEC^R^ strains was not affected (Fig. S4). Note that, on MH plates, strain MR271 grew much slower than the other strains, likely due to a burden imposed by the large DNA duplication. Therefore, mutations conferring MEC resistance also conferred resistance to A22.

### MEC^R^ mutants show different behaviors at the single-cell level

**Fig 5:**
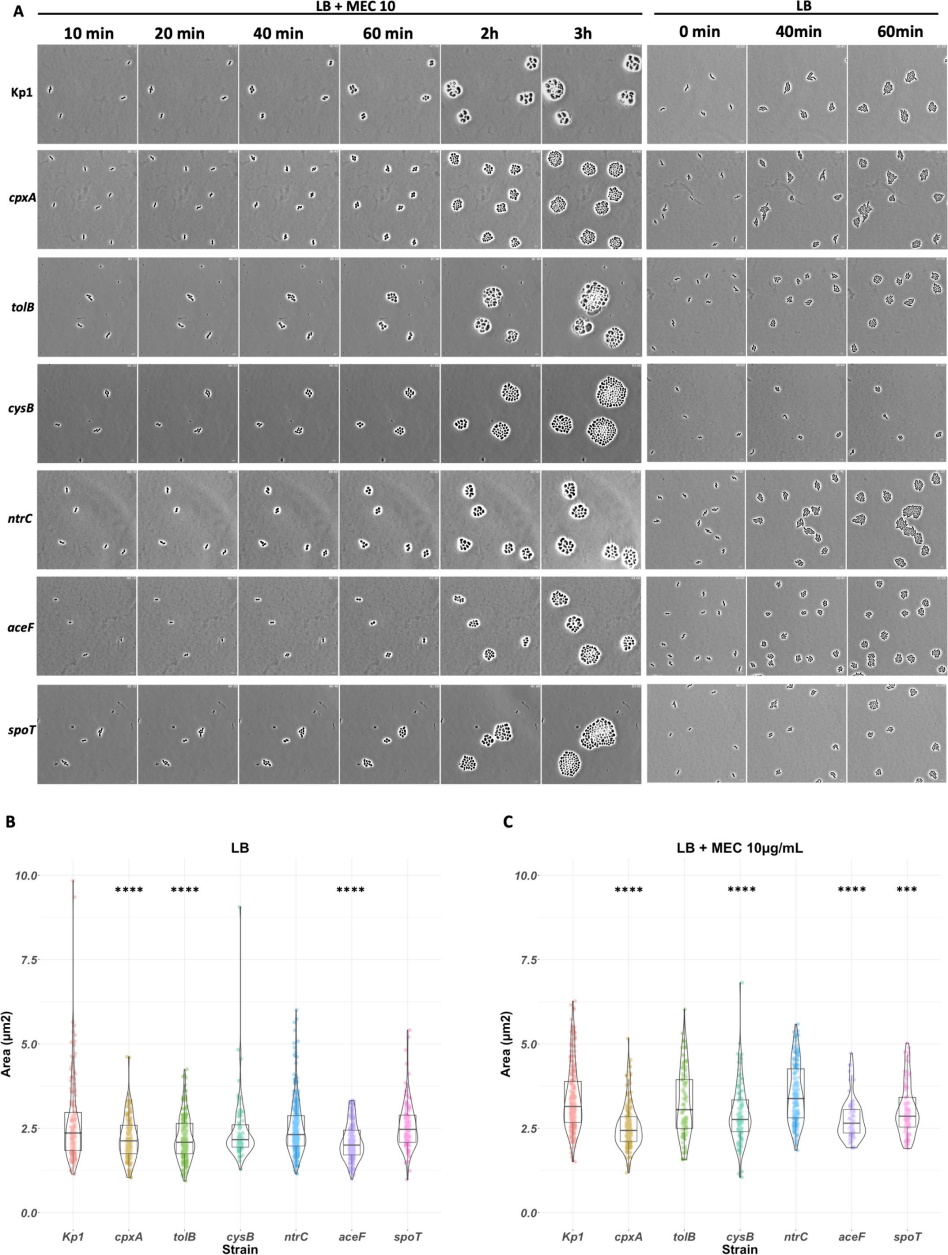
The impact of MEC on Kp1 and *aceF, cpxA, ntrC, tolB, cysB,* and *spoT* MEC^R^ mutants at the single-cell level. Three-hour time-lapse microscopy experiments were performed on LB agarose pads supplemented with or without 10 µg/mL MEC. Phase contrast observations were done on a Nikon inverted Ti microscope with images taken every 5 min with a sCMOS ORCA Flash 4.0 camera. (**A**) Images of the seven strains at 10, 20, 40, and 60 min, 2 and 3 h on LB+MEC, and at 0, 40, and 60 min on LB following inoculation. Scale bar = 5 µm. (**B and C**) Violin plot of the distributions of bacterium area at 40 min on LB pad (**B**) and on LB+MEC pad (**C**) with boxplots representing from top to bottom the third quartile, the median, and the first quartile. Picture analysis was done in Fiji (49) with the MicrobeJ plug-in (50). A Student’s test was performed to compare the area of mutants with Kp1. *P < 0.05; **P < 0.01; ***P < 0.005; and ****P < 0.001. Live-cell imaging data will be provided upon request.

**Fig 6:**
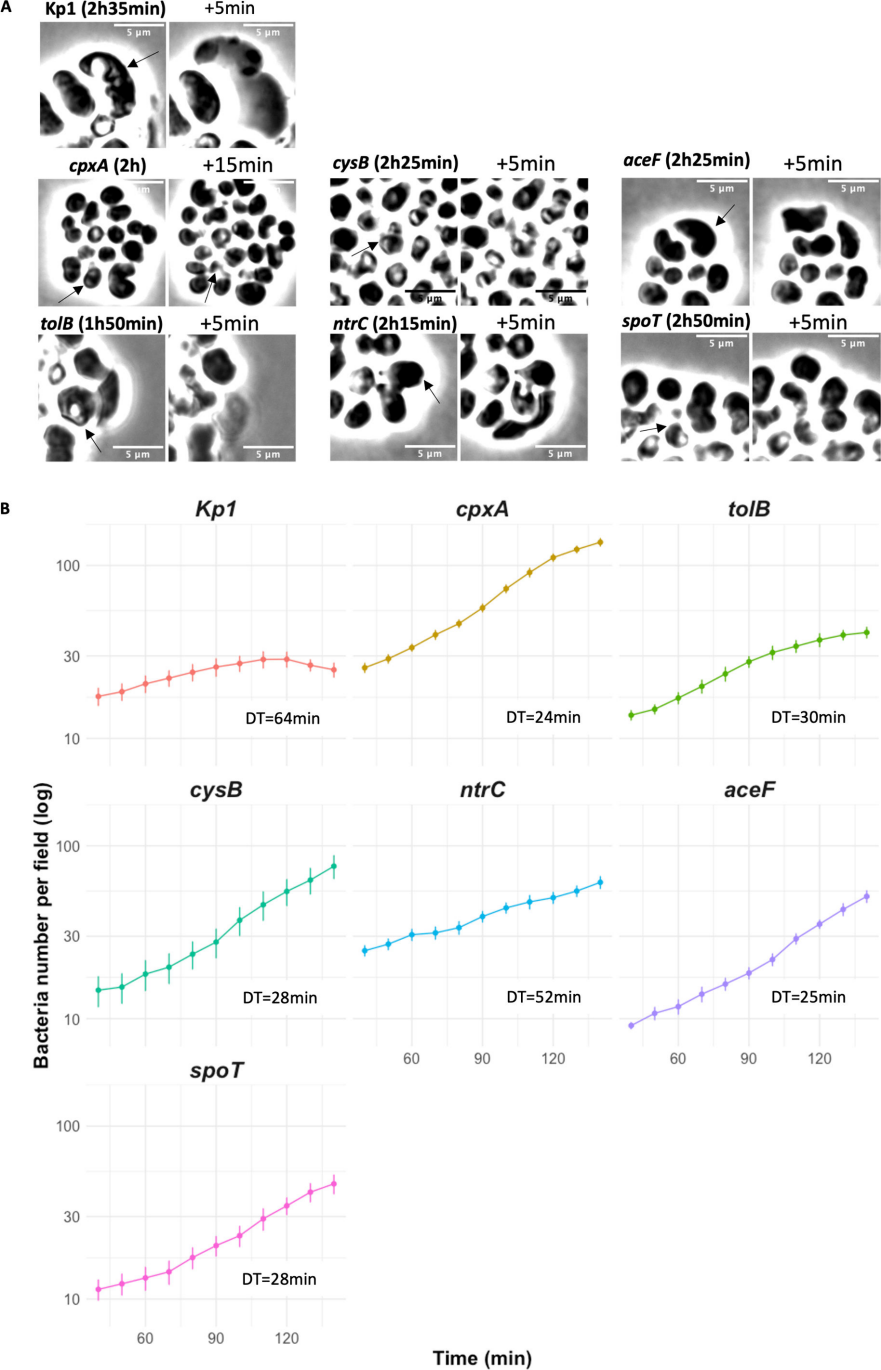
Bacterial growth and death as determined by time-lapse experiments. (**A**) Snapshots of Kp1 and *aceF*, *cpxA*, *ntrC*, *tolB*, *cysB,* and *spoT* MEC^R^ mutants at different time points of the 3-h time-lapse microscopy experiments in the presence of 10 µg/mL MEC as in Fig. 4. Ghost cells or leakages are pointed out by black arrows. Scale bar = 5 µm. (**B**) Bacterial multiplication of the seven strains in the presence of MEC is determined by counting cells every 10 min from 40 to 140 min. Each point represents the mean, and the error bars represent the standard error of at least eight different stacks. The points are represented in log scale, and the doubling time (DT) was calculated according to the slope of the mean of the eight stacks. Live-cell imaging data will be provided upon request.

Bacterial growth rate results from cell multiplication and death. Its measurement by following the OD_600_ is also affected by the bacterial cell shape. To further explore reasons for the growth defect in the presence of MEC, we analyzed the growth of Kp1 and of the six MEC^R^ mutants at the single-cell level by time-lapse microscopy. Bacterial growth phenotype was monitored on LB agarose pad in the absence or presence of 10 µg/mL MEC for 3 h (Fig. 5 and 6). In the absence of MEC, Kp1 multiplied during the 3 h of the experiments with a doubling time of approximately 20 min (Fig. 5A). When adding MEC, Kp1 turned into round-shaped bacteria with an increased area at 40 min after incubation of 3.4 µm^2^ compared to 2.7 µm^2^ in the absence of MEC (Fig. 5; Fig. S5). Then, Kp1 bacteria underwent a few abnormal cell divisions and eventually lysed for most of them (Fig. 6A). This resulted in a slow increase in Kp1 bacterial number (Fig. 5A; Fig. 6B).

The *aceF*, *tolB,* and *cpxA* mutant cells were significantly smaller on LB pads than Kp1 cells, with an average area of 2.1, 2.2, and 2.2 µm^2^ at 40 min post-inoculation compared to 2.7 µm^2^ for Kp1, respectively (Fig. 5A and B). In the presence of MEC, the six mutants also turned into round-shaped bacteria with increased area, indicating that MEC is still inhibiting PBP2 in these strains (Fig. 5A; Fig. S5). The *tolB* and the *ntrC* mutants showed an increase in cell size, like what was observed in Kp1 (Fig. 5C). They also experienced bacterial cell shape evolution reminiscent of Kp1, leading in some cases to cell death, with a more pronounced phenomenon for the *tolB* mutant. However, both mutants continued to divide. The four other mutants divided in the presence of MEC and formed microcolonies despite some bacterial shape deformation and rare events of cell death (Fig. 6A). All mutants showed an exponential increase in cell numbers, but with different doubling times (Fig. 6B). The *ntrC* mutant had the longest doubling time (52 min) compared to the other strains. The *tolB* mutant showed a slowdown during the last 40 min of the experiment. This slowdown resulted from the high cell death rate.

### Mecillinam resistance of mutants in amino acid metabolism and translation categories is RelA dependent

In *E. coli,* the *ftsZAQ* overexpression and MEC resistance can be triggered by the stringent response via an increased (p)ppGpp level (11, 14, 51). To identify RelA-dependent MEC resistance paths in *K. pneumoniae*, we deleted the *relA* gene in strain Kp1 (strain Kp1∆*relA*). We measured the MEC^R^ colony frequency in this mutant at different MEC concentrations (8/16/32/50/64/100/200 µg/mL) and selected MEC^R^ colonies that grew on 50/100/200 µg/mL MEC for genome sequencing, as we did for Kp1. Colonies appeared at a frequency similar to strain Kp1, except at the highest concentrations of 100 and 200 µg/mL, where frequencies were lower (Fig. S6). Among the 72 Illumina-sequenced colonies, 21% were mutated in the cell wall category (Table 2), similar to the 25% found in the *relA*+ background. Mutations affecting central carbon metabolism were enriched with 42% of the MEC^R^ mutants compared to 16% for *relA*+. Ten strains were mutated in the *aceEF* locus (pyruvate dehydrogenase) compared to four in the Kp1 background (Table 2). Conversely, the only MEC^R^ mutant (1%) identified in the ∆*relA* background that fell into the amino acid metabolism category was *asnP*, which encodes an asparagine permease. In contrast, 14% (*n* = 12) of the MEC^R^ mutants in the *relA*^+^ background belonged to this category. Only two (3%) MEC^R^ mutants in ∆*relA* were associated with the translation category, compared to 7% in *relA*+ (Table 2). In ∆*relA*, these two mutants carried mutations in *rlpB* (encoding the large ribosomal subunit protein L2) and *epmB*, whose product, together with EpmA, contributes to the β-lysylation of the translation elongation factor P. Notably, no mutants in tRNA synthetase genes were recovered in the ∆*relA* background. These findings suggest that the MEC^R^ phenotype associated with mutations in genes related to amino acid metabolism is RelA dependent, as is the case for most genes involved in translation.

**TABLE 2:** Distribution of mutations in the 72 sequenced Kp1∆relA MEC^R^ mutants

Category^*a*^	Gene	Function	Nbr^*b*^	Mutations^*c*^	Nb^*d*^	Kp1^*e*^
Cell wall (21%)	*cpxA*	Sensor protein, Cpx system	4	(CTG)_4→3_ nt502	3	17
				I95S	1	
	*tolA*	Inner membrane protein, Tol/Pal system	4	Q7^*^	1	3
				Q68^*^	1	
				Q167^*^	2	
	*tolB*	Periplasmic protein, Tol/Pal system	2	W426^*^	1	1
				∆1bp nt86	1	
	*tolQ*	Inner membrane protein, Tol/Pal system	4	S28F	1	0
				W32^*^	1	
				L19R	1	
				Q165^*^	1	
	*igaA*	Intracellular growth attenuator protein, RCS system	2	T145P	2	0
Amino acid metabolism (1%)	*ansP*	L-asparagine permease	1	Δ199 bp nt158	1	0
Central carbon metabolism (42%)	*aceE*	Pyruvate dehydrogenase complex subunit E1	8	S177F	1	2
				∆243bp nt581	1	
				2bp → TT nt1888	1	
				W579^*^	1	
				I648T	1	
				I570S	1	
				A766V	1	
				∆1bp nt426	1	
	*aceF*	Pyruvate dehydrogenase subunit E2	2	∆1bp nt1606	1	2
				∆620bp nt706	1	
	*lpdA*	Pyruvate dehydrogenase subunit E3	1	T124P	1	0
	*guaA*	GMP synthase	2	H181Q	1	0
				C202F	1	
	*ubiF*	Ubiquinone and menaquinone biosynthesis	1	∆1bp nt896	1	0
	*ubiH*	Ubiquinone and menaquinone biosynthesis	3	(G)_9→8_ nt27	3	3
	*cydA*	Cytochrome bd-I ubiquinol oxidase subunit 1, aerobic respiration	4	+T nt110	2	0
				W118^*^	1	
				IS903 nt123	1	
	*hemA*	Glutamyl-tRNA reductase	1	V289D	1	0
	*lipA*	Lipoyl synthase	3	D140A	1	2
				∆13bp nt865	1	
				K118^*^	1	
	*ndh*	NADH: quinone oxidoreductase type II	1	Q332R	1	0
	*gnd*	Pentose phosphate pathway	1	+C nt375	1	0
	*ackA*	Acetyl-CoA biosynthesis	4	(ACGCCCAGT)_1→2_ nt1044	1	0
				R91S	1	
				∆1bp nt959	1	
				C225Y	1	
	*csrA*	Carbon storage regulator	1	V25G	1	0
Translation (3%)	*epmB*	L-lysine 2,3 amino-mutase	1	IS903 nt139	1	1
	*rplB*	50S ribosomal protein L2	1	S118P	1	0
Intergenic mutations (17%)	*yrdA/rRNA 16S*	*yrdA*: carbonic anhydrase	1	T→A nt −247	2	0
	*dauA/prs*	*dauA*: C4-dicarboxylic acid transporter *prs*: ribose-phosphate pyrophosphokinase	8	C→A nt −120	8	5
	*ENJAKFCB_04041, thiI, xseB, ispA*	tRNA and lipid metabolism	1	∆2,737bp nt4242023	1	0
	*rutG/ ENJAKFCB_03303*	*rutG*: putative pyrimidine permease	1	G→A nt12	1	0
	*ENJAKFCB_00197/yiaF*	Hypothetical proteins	1	C→A nt −241	1	0
	*msrP, csrD, mreB*	*csr*: small RNA degradation; *mreB*: rod system	1	∆ 3,179bp nt473827	1	0
Others (32%)	No mutation		7		7	25
	720 proteins		9	Amplification	9	6
	64 phage proteins		8	Amplification	8	0
	*ENJAKFCB_00013*	Hypothetical protein	1	(C)_8→9_ nt351	1	0
	*ybaP*	TraB protein family	1	Q94P	1	0
	*ENJAKFCB_01639*	Hypothetical protein	1	IS903 nt773	1	0
				IS903 nt781	1	
	*ENJAKFCB_04857*	Hypothetical protein	1	∆361bp nt −63	1	0
	*bioB*	Biotin synthase	1	Y258C	1	0

^*a*^ Percentages are calculated based on the total number of sequenced mutants, as some mutants harbored several mutations. The sum of the percentages is higher than 100%.

^*b*^ Number of isolates mutated in the gene.

^*c*^ Asterisk means non-sense codon.

^*d*^ Number of isolates with the same mutation in the same gene.

^*e*^ Number of isolates mutated in the same gene in Kp1 (Table 1).

**Fig 7:**
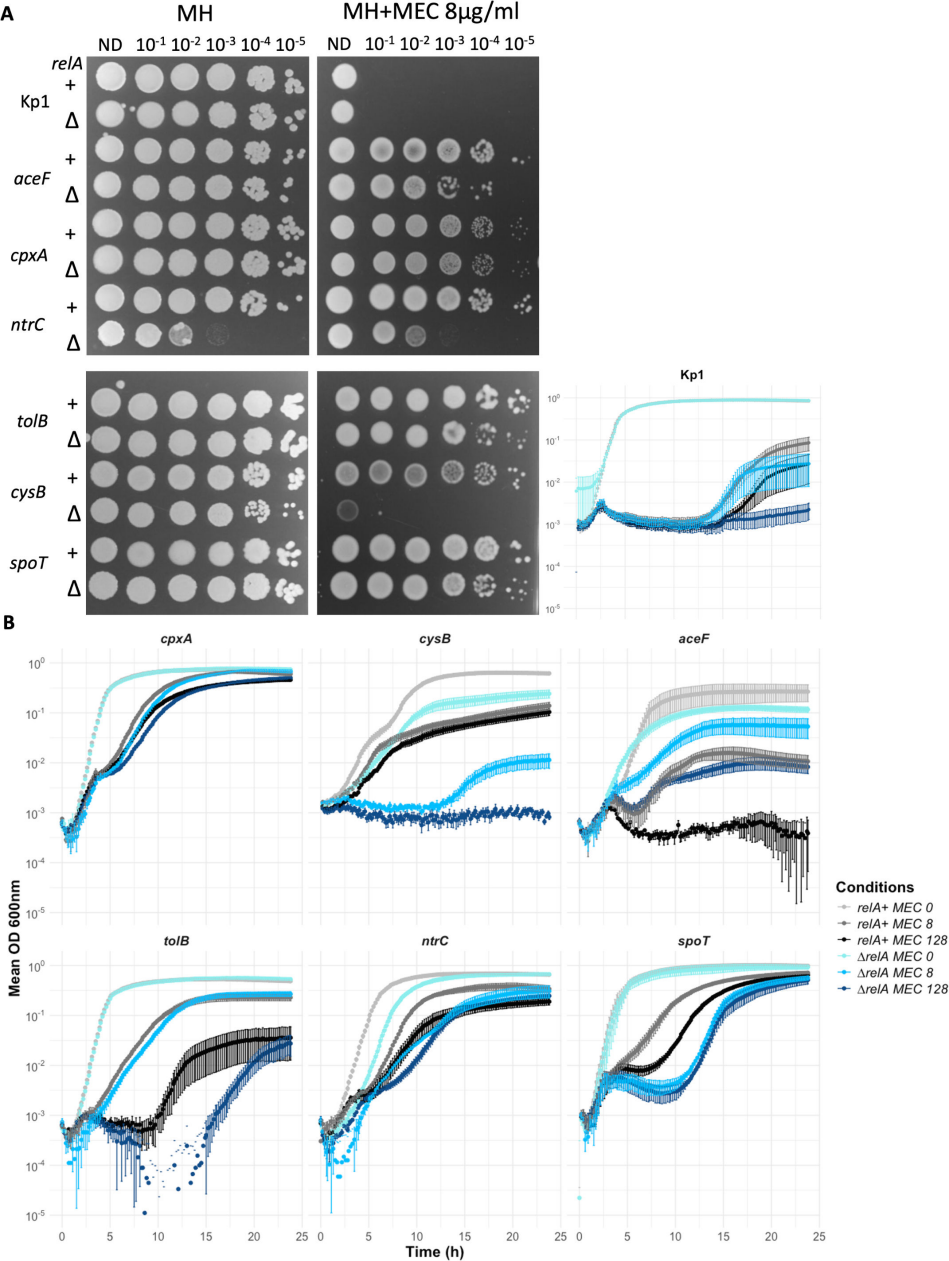
Impact of *relA* deletion on MEC susceptibility of Kp1 and *aceF, cpxA, ntrC, tolB, cysB,* and *spoT* MEC^R^ mutants on MH plates and in broth. (**A**) Susceptibility testing on a plate. Bacterial cultures were diluted at an OD_600_ of 0.2 and serially diluted as indicated on top of each image and spotted on MH agar plates supplemented with or without 8 µg/mL MEC. (**B**) Twenty-four-hour growth curves of the *∆relA* mutants derivative of Kp1 and of *aceF, cpxA, ntrC, tolB, cysB,* and *spoT* MEC^R^ mutants in MH supplemented with MEC at 0 (light colors), 8 (colors), or 128 µg/mL (dark colors). Growth curves of Kp1 mutants are in gray, and curves of the ∆*relA* derivatives are in blue. Bacterial culture diluted at an OD of 0.001 was distributed in a 96-well plate with MH supplemented with or without 8 or 128 µg/mL MEC. The OD_600_ was measured for 24 h every 11 min. For both experiments, as the *spoT*∆*relA* strain harbored an additional mutation, we compared it to a *spoT* strain (MR575) sharing the same additional mutation. Each point represents the mean of at least three replicates, and the error bars represent the standard error. The overnight culture of the *cysB* mutant and its ∆*relA* derivative was performed in LB supplemented with 0.5 mM L-cysteine.

To confirm these observations, we tested the epistatic relation between *relA* and the six previously studied MEC^R^ mutants (in *cpxA*, *tolB*, *cysB*, *ntrC*, *aceF,* and *spoT*). In the first step, we tested the amino acid auxotrophy of these mutants. The *aceF* and *cysB* mutants did not grow on minimal medium plates. The *cysB* mutant, like the *cysE* mutant, is also resistant to MEC and does not grow on minimal medium. However, it grew on minimal medium plates complemented with L-cysteine (0.5 mM) (Fig. S7). Therefore, these two strains were auxotrophic to L-cysteine. We next constructed ∆*relA* derivatives of the six mutants and analyzed the MEC^R^ phenotype by spotting serial dilutions on MH agar plates supplemented with or without 8 µg/mL MEC. Compared to their *relA*+ counterparts, the *relA* deletion led to MEC susceptibility of the *cysB* mutant (Fig. 7A). Growth of the *ntrC*∆*relA* strain (amino acid metabolism category) was also affected with or without MEC (Fig. 7A). We confirmed these results by following growth of the strains in liquid culture in the presence of MEC (8 and 128 µg/mL). The *cysB*∆*relA* strain could only grow after 12 h of incubation, like the parental Kp1 strain, and the *ntrC∆relA* mutant showed an affected growth in the presence or absence of MEC (Fig. 7B). The *spoT*∆*relA* mutant showed a longer lag time in the presence of MEC at both MEC concentrations. At 8 µg/mL, the growth curves were similar between *cpxA* and *tolB* mutants and their ∆*relA* derivatives, but the *tolB*∆*relA* mutant showed a longer lag phase of 15 h at 128 µg/mL, similar to what was observed for Kp1. Note that the *aceF*∆*relA* strain grew better in the presence of MEC than its parental *aceF* mutant (Fig. 7B).

### Clinical relevance of the *cpxA* mutation

In Kp1, the most frequently observed mutation (*n* = 16 out of 87 sequenced MEC^R^ colonies) was the deletion of one CTG leucine codon among four consecutive CTG codons starting at position 165 of the *cpxA* gene. In Kp1∆*relA,* 3 out of 72 MEC^R^ colonies showed the same mutation. We, therefore, asked whether this mutation could be detected among clinical isolates by searching for this exact mutation among the 4,410 *K. pneumoniae* complete genomes available at NCBI on 15 July 2025. Fifteen showed this mutation (Table S11). In most cases, these GenBank entries refer to antibiotic resistance of clinical isolates. Seven were associated with publications referring to *in vivo* evolution or carbapenem resistance (Table S11). For example, the loss of this leucine codon was observed during the evolution toward imipenem resistance of strain ZRKP04, which expresses the carbapenemase KPC-33 from strain ZRKP01 (52). Therefore, this mutation might contribute to an increased resistance among clinical isolates.

## DISCUSSION

Mecillinam is a narrow-spectrum β-lactam used opportunistically to treat uncomplicated UTIs. It targets a broad range of bacterial species, including *K. pneumoniae* (29). Here, we performed an in-depth characterization of the MEC resistome in *K. pneumoniae*. First, we observed that MEC^R^ colonies grew faster and at higher concentrations than in *E. coli* MG1655, even though the two parental strains showed the same MEC MIC. These differences were due to the intrinsic β-lactamase SHV-11 and were confirmed with other *bla*_SHV_ genes. It was not due to an increased tolerance to MEC, as we observed similar time-kill curves at different MEC concentrations for Kp1∆*bla*_SHV-11_ and Kp1 (Fig. S1). The relative rate of hydrolysis and the apparent affinity for MEC were determined using a crude extract of SHV-3-producing *E. coli* (53). SHV-3 binds MEC with a low affinity (100-fold lower than for ampicillin) but has a high hydrolysis rate (as for ampicillin). Similar hydrolysis properties of MEC by other SHV β-lactamases would explain the growth of *K. pneumoniae* strains and *E. coli* expressing *bla*_SHV-11_ MEC^R^ mutants at high MEC concentrations despite the low MIC (0.22 and 0.125 µg/mL) of the parental strains. Indeed, according to these hydrolysis parameters, the SHV enzyme would only hydrolyze MEC at high concentrations, allowing the growth of MEC^R^ mutants at concentrations higher than 200 µg/mL. To our knowledge, this is the first report of SHV β-lactamases as potentiators of *K. pneumoniae* resistance.

The MEC resistome has been investigated in *E. coli* by using different experimental strategies (18–21). A high-throughput characterization was achieved by using TnSeq on three *E. coli* strains (MG1655, MG1655 ∆*relA,* and MG1655 ∆*csrB*) expressing *ftsZ* on a monocopy plasmid (20). To describe the MEC resistome in *K. pneumoniae*, we Illumina-sequenced mutant strains selected on MEC-supplemented MH plates. Selection was performed on three strains: a recently isolated pan-susceptible strain, Kp1, and two derivatives deleted for *bla*_SHV-11_ and *relA,* respectively. As previously shown in *E. coli,* we isolated strains mutated in a broad range of functions, which could be classified into four main functional categories: carbon metabolism, cell wall, translation, and amino acid metabolism. Most genes mutated in our study were described in the *E. coli* MEC resistome, except the *cpxA* gene. TnSeq would mainly detect loss-of-function phenotypes, but the *cpxA* mutations selected in Kp1 presumably resulted in a gain of function. Supporting this hypothesis, the inactivation of the *cpxA* gene (strain Kp1 ∆*cpxA::kanaR-tse2*) has no effect on MEC MIC (Table S1). We confirmed experimentally that the predicted causative mutation was responsible for the resistance phenotype for four mutants (*cysB*, cpxA, *ntrC,* and *tolB*). We did not succeed in reintroducing the *aceF* and *spoT* mutations in the parental strain. However, the *aceF* gene was found to be mutated in two MEC^R^ Kp1 and two MEC^R^ Kp1∆*relA* mutants. The other subunit of the pyruvate dehydrogenase, encoded by *aceE,* was mutated in two MEC^R^ Kp1 and in eight MEC^R^ Kp1∆*relA* mutants. *aceF* was also described as a MEC resistance allele in the Tn-Seq of Lai et al*.* (20)*. spoT* is an essential gene in *E. coli* (54). However, we selected three different MEC^R^ Kp1 strains mutated in this gene. *spoT* MEC^R^ mutants were also reported in *E. coli* (19). Thus, it is likely that these mutations were responsible for the MEC^R^ phenotype observed*.* Similarly, the isolation of different mutations in other genes (Table S5) that are also found in the *E. coli* MEC resistome (18–20) strongly supports their causality in the MEC^R^ phenotype.

We did not detect a MEC^R^ strain mutated in *mrdA*, the structural gene of the MEC target, PBP2. An *E. coli* MEC^R^ mutant in *mrdA* (D389G) was isolated by similarly plating a susceptible strain on plates containing different concentrations of MEC (19). This *mrdA* mutant showed a strong growth defect, and *mrdA* mutants were not detected in clinical isolates (19). The frequency of MEC^R^ mutants in *E. coli* is lower than in Kp1. Therefore, a combination of a high fitness cost of *mrdA* mutations and the high frequency of selection of other mutations or genome amplification might have outcompeted mutations in *mrdA,* explaining their absence in our collection.

In the three strains, the most frequent genetic event was an 802 kb-long duplication bracketed by two rRNA operons. This region encompasses the *dcw* locus, including the *ftsZ* gene. In their extensive study of MEC^R^ mutants in *E. coli*, Vinella et al. (51) showed that a significant proportion of mutants were unstable, and this instability was RecA dependent. MEC resistance in unstable mutants was also shown to be (p)ppGpp independent and to result from a large (>250 kb) chromosomal duplication, including *ftsZ* (51). *E. coli ftsZ* is also located between two ribosomal operons, *rrnE* and *rrnH,* separated by 698 kb. As we have observed in Kp1, Vinella et al.’s data (51) in *E. coli* are best explained by a duplication of the chromosomal region between *rrnE* and *rrnH* by replication slippage between the two ribosomal operons. This duplication in *K. pneumoniae* led to a fitness cost, and the resulting resistance phenotype is unstable, fulfilling the definition of heteroresistance (40). Expression of *ftsZ* on a monocopy plasmid was shown to be not sufficient to induce MEC resistance (12, 51), contrasting with our results. However, the duplication involves a region of 720 genes, including the whole *dcw* locus and *mrcB* encoding PBP1B. Some of these genes, like *mrcB*, whose upregulation contributes to MEC resistance in a *cysB* mutant of *E. coli* (11), might also contribute to the resistance phenotype. Possibly due to their instability, such duplications have not been reported in clinical isolates, although they might be a first step toward resistance.

The second most frequent genetic event was the loss of a CTG leucine codon in a tandem repeat of four CTG codons in *cpxA* (codons 165–168). It was mutated in 17 out of 88 Kp1 MEC^R^ mutants and in three Kp1∆*relA* mutants. This mutation likely resulted from a slippage of the DNA polymerase during chromosome replication. We can assume that such an event is reversible, reminiscent of a phase variation phenomenon. In *E. coli,* the four leucine residues are conserved, but the codons are different, with CTA TTA CTG CTG, a sequence not prone to DNA replication slippage, possibly explaining the absence of this mutation among *E. coli* MEC^R^-resistant strains. In *K. pneumoniae* and *E. coli*, a mutation in this gene, Y144N in the periplasmic sensor domain, was described as contributing to resistance to ampicillin, ceftazidime, imipenem, and fosfomycin (55). The loss of a CTG leucine codon in *cpxA* affects the transmembrane domain downstream of the periplasmic sensor domain and might activate it similarly. Hence, this mutation could lead to an increased expression of the envelope stress response (56), including the expression of the LD transpeptidase LdtD, leading to a remodeling of the peptidoglycan (57) responsible for escaping the MEC bactericidal effect. We identified 15 out of 4,410 *K. pneumoniae* complete genomes deposited at the NCBI showing the same loss of a CTG leucine codon. In several cases, the strain was isolated in the context of β-lactam resistance studies, suggesting that this mutation can also be selected during treatment.

In *E. coli*, the large diversity of mutations leading to MEC resistance has been classified according to different criteria. First, in 2000, Vinella et al*.* (51) determined if the MEC resistance was (p)ppGpp dependent, and more recently, Lai et al*.* (20) defined if it was RelA or Rcs dependent. In Kp1∆*relA*, we observed a drop of mutants in genes involved in translation and amino acid metabolism. Deletion of the *relA* gene abolished the resistance phenotype of the Kp1 *cysB* E136* mutant. Therefore, MEC resistance of this mutant and likely also of the *cysE* mutant could be linked to the induction of the stringent response, likely explained by their L-cysteine auxotrophy (Fig. S7). In *E. coli*, the deletion of both *relA* and *spoT* was found not to restore MEC susceptibility in a ∆*cysB* strain, and it was proposed that MEC resistance occurred in response to the oxidative stress elicited in this mutant (11). The reasons for this different behavior between *E. coli* and Kp1 are unclear. It could be that, in *E. coli* but not in Kp1, MEC resistance linked to the oxidative stress somewhat masks an effect through the stringent response. Alternatively, differences in transcriptional or metabolic networks in *K. pneumoniae* might be responsible for these observations. Finally, we cannot rule out that the different behavior might be due to the synthesis of the N-terminal domain encompassing the helix-turn-helix DNA-binding motif in the Kp1 *cysB* mutant studied here (58). This domain might have a different effect on the bacterial physiology compared to the absence of the regulator in *E. coli*.

The deletion of *relA* also negatively affected the growth of the *spoT* and *tolB* mutants in the presence of MEC, likely through different mechanisms (Fig. 7). The T162M mutation in *spoT* is in the hydrolase domain, probably leading to an increased (p)ppGpp level, which would be partially reversed by the loss of RelA. In these two strains, MEC resistance would be partially (p)ppGpp dependent, reminiscent of what is proposed by Vinella et al. (51). Conversely, the growth of the *aceF∆relA* mutant in the presence of MEC was less impacted than the growth of the *aceF* mutant. We hypothesized that in mutants defective in the pyruvate dehydrogenase, the impaired tricarboxylic cycle would slow down the bacterial growth, as observed in the absence of MEC. Under MEC exposure, the stringent response might be activated in the *aceF* mutant, impacting its growth more than in its ∆*relA* derivative.

In *E. coli*, MEC triggers a metabolic futile cycle by inducing the newly synthesized PG degradation (12) and by shifting the central carbon metabolism from glycolysis to PG biosynthesis, leading to “energy-starved bacteria” (59). As in *E. coli,* we observed a broad diversity of MEC^R^ mutants that bypass the need for PBP2. The six mutant strains we have in-depth analyzed and the strain showing a duplication of the *dcw* locus were resistant to the *mreB* inhibitor A22. Therefore, the elongasome is no longer essential in these mutants. The growth characteristics of MEC^R^ *K. pneumoniae* mutants remained largely unknown. Here, we characterized the growth of the six mutant strains in the presence of MEC under three different laboratory conditions: (i) in liquid, by following the OD_600_ and computing lag time, growth rate, and maximum OD reached; (ii) on plates by analyzing the inhibition halo; and (iii) at the single-cell level by time-lapse microscopy. Under these three conditions, growth was deeply affected by MEC, suggesting that the futile cycle was not suppressed. Growth parameters in the presence of MEC of the mutated strains were affected differently and were not correlated with a functional category. Net bacterial growth is a result of cell division and cell death. In liquid culture, according to the turbidity at 600 nm, *tolB* and *aceF* mutants showed similar doubling times in the presence of MEC (8 µg/mL): 40.1 and 43.4 min, respectively (Fig. 4B). But time-lapse experiments revealed two very different behaviors. In the *tolB* strain, an increase in cell size compensated for a high mortality rate in the OD_600_ quantification, whereas in the *aceF* strain, the cells remained smaller than those of Kp and experienced rare cell death. In this regard, the peculiar growth around the E-test inhibition halo of the *tolB* mutant might result from rapid growth and death in the presence of MEC (Fig. 3). The Eagle effect observed for the *aceF* mutant suggests an optimal MEC concentration where the mutant could grow. In liquid culture, the growth of this mutant was less reproducible due to the variability of the phenotype. Therefore, the six MEC^R^ mutations in different functional categories disturb different metabolic and/or stress pathways, allowing growth in the presence of MEC by bypassing the need for PBP2 but only partially alleviating the impact of the futile cycle.

Antibiotic resistance in *K. pneumoniae* is one of the most urgent threats to hospital hygiene. However, knowledge of the resistance mechanisms involved in this species lags what is known in *E. coli* despite its ability to become much more resistant than the model species. Here, we showed that the class A β-lactamase SHV contributes to reaching high levels of resistance to MEC. In *E. coli*, MEC^R^ mutants have been characterized according to their MIC or the fitness cost they impose on the bacterial cell in the absence of the β-lactam. Here, we investigated the MEC action on the growth and death of six *K. pneumoniae* MEC^R^ mutants, which are all deeply affected but in very different ways. We demonstrated a diversity of responses consistent with a MEC killing mechanism that is more complex than inhibition of PBPs alone. These MEC^R^ mutants were selected under *in vitro* conditions. However, we did not investigate their fitness in urine or other relevant biological fluids to predict their selection during treatment, as it was done for *E. coli* (60). Among clinical isolates, we identified strains showing the same Leu codon loss in CpxA; however, they were not identified as MEC^R^. The characterization of resistant strains escaping MEC treatment would be needed to identify the mutations selected in patients. This study aims to give a global picture of the MEC resistome in *K. pneumoniae* as a tool for other studies on β-lactam resistance mechanisms. It also emphasizes the need for further work on the antibiotic mode of action and resistance in *K. pneumoniae* to improve our capacity to better manage this threat.

## MATERIALS AND METHODS

### Bacterial strains and growth conditions

Characteristics of the strains used in this study are summarized in Table S1. Kp1 from the Kremlin Bicêtre strain collection was isolated in 2018 from a stool sample from an anonymous patient. Kp1 carries a chromosomal *bla*_SHV-11_ gene and no acquired resistance gene. Strain derivatives of *K. pneumoniae* strain Kp1 or of *E. coli* MG1655 (61) are listed in Table S1. Bacterial strains were grown in lysogeny broth (LB) or in Mueller-Hinton (MH). Bacteria isolation and spotting were performed on MH agar plates. Medium was supplemented, as indicated, with chloramphenicol (Cm, 20 µg/mL), kanamycin (Km, 40 µg/mL), zeocin (Zeo, 35 µg/mL), A22 (10 or 20 µg/mL), mecillinam (MEC, at the indicated concentrations), potassium clavulanate (2 µg/mL), and L-cysteine (0.5 mM).

Auxotrophy was tested by analyzing growth on M9 minimal medium plates. Strains were grown overnight on LB agar and streaked on M9 agar supplemented with glucose 0.4% for 24 h at 37°C. If no colonies appeared after 24 h of incubation, auxotrophy was tested by complementing the medium with the suspected amino acid. *cysB* and *cysE* mutants were streaked in parallel on glucose 0.4% M9 agar supplemented with or without 0.5 mM L-cysteine (Sigma-Aldrich) for 24 h at 37°C.

### MEC^R^ colony frequency quantification

Overnight precultures in LB were diluted 5,000 times in LB and grown at 37°C at 180 rpm until they reached an OD_600_ of approximately 2. These cultures were diluted 30 times and 10^6^ times in physiological water. MEC^R^ CFUs were quantified by plating 100 µL of the 30× dilutions on MH agar supplemented with MEC at different concentrations using the automatic plater easySpiral (Interscience). Total CFUs were counted by plating 100 µL of the 10^−6^ diluted suspension on MH agar. Colonies were counted using the Scan4000 (Interscience) at different time points of incubation at 37°C as indicated. Spontaneous MEC^R^ colony frequency was calculated by dividing the CFU/mL on MEC plates by the CFU/mL on MH. Colonies from different plates were isolated at the 19-h time point on MH plates and stored at −80°C with 25% glycerol for further analysis and whole-genome sequencing.

### Minimal inhibitory concentration determination, spotting assay, and growth curves

MEC MIC was determined by E-test. Strains were grown overnight in LB medium at 37°C at 180 rpm and diluted to reach an OD_600_ of 0.1 and further diluted 1:100 in MH. The flooding method was selected over swab streaking as it provides more reproducible results. The MEC E-test strip (bioMérieux) was applied to the agar plate. E-tests were read according to the manufacturer’s instructions after 16 h of incubation at 37°C. Comparison of the susceptibility of the MEC^R^ mutant strains and their ∆*relA* counterparts was performed by spotting 4 µL serial dilutions of stationary phase cultures on MH plates containing 8 µg/mL MEC. The susceptibility to A22, an MreB inhibitor, was similarly assessed by spotting dilutions on MH plates containing 10 or 20 µg/mL A22 (Sigma-Aldrich).

Bacterial growth in MH broth supplemented with MEC at different concentrations was analyzed by using an automatic plate reader (Tecan Infinite M Nano). Plates (96-well) were filled with 50 µL of MH supplemented with MEC (Cayman Chemical) to reach final concentrations of 2/4/8/16/32/64/128 µg/mL or only at 8 and 128 µg/mL for ∆*relA* derivatives. Fifty microliters of the diluted solution of bacteria at OD_600_= 10^−3^, as described for E-test, was added to each well, and the plate was incubated at 37°C under orbital shaking at 198 rpm for 24 h with an OD_600_ measurement every 11 min.

### Time-kill experiments

To compare the kinetics of the MEC killing of Kp1 and Kp1∆*bla*_SHV-11_, we performed time-kill experiments in droplets (62). Starting from overnight cultures, we inoculated the two strains in MH broth to reach the exponential phase. After normalizing the OD_600_ at 0.2, cultures were 50-fold diluted in 1 mL of MH supplemented with MEC at concentrations ranging from 0.0625 to 1 µg/mL or from 1 to 16 µg/mL in a 24-well plate. Plates were incubated at 37°C under agitation. Every 30 min for 3 h 30 min, 100 µL of each well was transferred to a 96-well plate and 1/5 serially diluted from non-diluted to 5^−7^. Using a replicator, we transferred one droplet from each well to two square MH plates to obtain duplicates. After an overnight incubation at 37°C, colony-forming units (CFU) were counted. The weighted average of each condition for both technical duplicates was calculated.

### Time-lapse microscopy on agarose pad

To compare the growth of the six mutants at the single-cell level, we performed time-lapse microscopy on agarose pads. Pads were made with LB 1.2% agarose supplemented with or without MEC at 10 µg/mL. Overnight cultures were diluted to start an exponential growth, and 1.2 µL of these cultures was deposited on the pad. One pad had four different droplets. Phase contrast observations were done on a Nikon inverted Ti microscope with a sCMOS ORCA Flash 4.0 camera. Snapshots of the seven strains were taken every 5 min on at least eight different stacks for 3 h at 37°C. Picture analysis was done by using Fiji (49) and its plug-ins MicrobeJ (50). Bacteria that were disappearing from the contrast, which showed leakage or that contained vesicle-like objects, were considered ghost cells and were not counted as growing bacteria.

### Gene deletion and allele replacement in strain Kp1

Kp1∆*bla*_SHV_, Kp1∆*mrdA_2,* and Kp1∆*relA* are Kp1 knockout derivatives that were constructed by homologous recombination mediated by the λ Red recombinase (63). Briefly, the kanamycin resistance cassette KanaR from an in-lab plasmid (IRC178; Table S12) was amplified by PCR, using the Q5 polymerase (NEB), with the two primer pairs MR35/36 and MR25/26 (Table S13) flanked with the sequences upstream and downstream of *mrdA* and *relA,* respectively. For Kp1∆*bla*_SHV_, the zeocin resistance cassette ZeoR from an in-lab plasmid was amplified as above with primers MR55 and MR56 (Table S13). The PCR reaction mix was digested by the restriction enzyme *Dpn*I to eliminate plasmid DNA. The PCR product was purified by using a Nucleospin column (Macherey-Nagel) and electroporated into the Kp1 strain carrying plasmid p15Red expressing the λ Red recombinase (Table S12). Transformants were selected on LB agar supplemented with Km or Zeo. Gene disruptions were verified by PCR using flanking primers (Table S13). The p15Red plasmid was next cured by plating the transformants on LB agar without NaCl and supplemented with 5% sucrose to activate killing by the levansucrase encoded by p15Red. Colonies were streaked on LB and LB supplemented with Cm to identify those that have lost p15Red. The absence of additional mutations was checked by Illumina sequencing.

For reconstructing the four *cpxA*, *tolB*, *ntrC,* and *cysB,* we first obtained knockout derivatives with the introduction of a kanamycin resistance cassette associated with the *tse2* toxin gene (Kmtse), as described above. We then amplified the mutated locus from the *cpxA*, *tolB*, *ntrC,* and *cysB* mutants, and by homologous recombination, we replaced the truncated gene with the mutated allele. We were unable to perform this first step for *spoT*, which is essential in *E. coli* (54) and likely also in *K. pneumoniae*. The counterselection was made on minimal 63B1 media supplemented with rhamnose 0.2% to induce the toxin (and L-cysteine for the ∆*cysB* mutant). The intermediate strain mutated in *aceF* grew very poorly, and we were unable to introduce the mutated allele by recombination. We selected three independent knockout mutants to perform three homologous recombination events per allele in parallel (two for *ntrC*). We validated the introduction of the mutated allele by PCR and Sanger sequencing. Primers used in these experiments are listed in Table S13 (IRC1153–IRC1172).

### Plasmid construction

The paGIBAC_SHV plasmid (Table S12) was constructed by Gibson Assembly (64) using a commercial kit (New England Biolabs) and following the manufacturer’s instructions. Primers overlapping with each other (Table S13) were designed with NEBuilder (New England Biolabs). *bla*_SHV-11_ and the upstream sequence containing its native promoter (65) were amplified using Kp1 chromosomal DNA and primers MR97 and MR98 (Table S13). The monocopy plasmid paGIBAC1 (37) (Table S12) was amplified by reverse PCR using paGIBAC1 plasmid DNA as a template and primers MR95 and MR96 (Table S13). PCR products were purified on Nucleospin columns (Macherey-Nagel) and quantified by Qubit 2.0 (Invitrogen). The Gibson reaction with an insert/vector ratio of 3:1 was performed at 50°C for 15 min. The reaction product was introduced into *E. coli* XL1-blue (Table S1) ultracompetent cells (Agilent) by heat shock transformation. Transformants were selected on LB supplemented with Cm, and the presence of plasmid was checked by PCR using primers MR105 and MR106 (Table S13). Plasmids were extracted with the Plasmid kit (Nucleospin, Macherey-Nagel) and sequenced (Whole Plasmid Sequencing, Eurofins). The plasmid paGIBAC_SHV was then electroporated into Kp1∆*bla*_SHV-11_ and MG1655 strains. The empty plasmid paGIBAC used as a control was constructed from paGIBAC_SHV by reverse PCR, ligated with the T4 DNA-ligase (New England Labs, M0202S), and electroporated into Kp1, Kp1∆*bla_SHV-11_*, and MG1655 strains.

### Whole-genome sequencing and mutation identification

Total bacterial DNAs were extracted from stationary phase cultures by using the Qiagen Blood and Tissue DNeasy Kit. The Kp1 strain was fully sequenced and used as the reference sequence by combining Illumina sequencing and PacBio long-read sequencing. Long-read PacBio sequences were assembled with hybrid-SPAdes version 3.15.5 for hybrid assembly of short and long reads (66). Annotation was performed by Prokka (67). Sequencing reads, assemblies, and annotations have been deposited at EBI under the project number PRJEB88497 (accessions are listed in Table S1). Illumina sequencing libraries were prepared with NEBNExt Ultra II FS DNA Library Prep Kit, and sequencing was performed with the NextSeq 500 or 2000 sequencing platforms. Short-read Illumina sequences were aligned to the Kp1 reference sequence with Breseq 0.35.7 (68) to identify SNPs, deletions, insertions, or amplifications. To check if new junctions detected by Breseq resulted from duplication, inversion, or insertion, read coverage was visually analyzed using Tablet version 1.21.02.08 (69). To identify large duplications undetected in the “new junctions” category by Breseq, we visually analyzed the global depth coverage of the chromosome according to the Breseq depth coverage output. In cases where a >1.8-fold jump was observed, depth coverage was checked by using Tablet. IS insertions were checked with ISFinder (70).

Annotation of the mutated gene was determined by combining the Prokka annotation (67), the annotation of the closest homologs in the SwissProt or TrEMBL database as determined by Blast in Uniprot (71), and the ortholog gene annotation in the NCBI reference strain Kp HS 11286 (CP003200.1). The mutated genes were assigned to functional categories, thanks to UniProt and EcoCyc: cell wall, amino acid metabolism, central carbon metabolism, translation, and others.

The loss of one leucine codon of the four CTG codons (positions 165–168) among the 4,410 complete *K. pneumoniae* genomes available at the NCBI was evaluated via tBLASTN on 15 July 2025.

### Statistical analysis

Statistical analyses were made with RStudio (version 4.4.1) by using the R packages (“tidyverse,” “dplyr,” “ggplot2,” “emmeans,” “performance,” and “ggstatsplot”). For comparison of the MEC MIC, we performed a Wilcoxon test. For analysis of growth parameters and colony appearance rate, we performed a two-way or three-way ANOVA, respectively, with raw data or the log, depending on their distribution. If the test was significant, we did a multiple comparison with Tukey or Šidák methods, depending on the number of conditions compared. For the time-lapse microscopy, we used a Student’s test to compare the mutants to the Kp1 strain. For all these analyses, we have a risk of 5%.

## SUPPLEMENTAL MATERIAL

Supplemental Material aac.01207-25-s0001.pdf Additional supplemental tables and Fig. S1 to S7.

Table S1 aac.01207-25-s0002.xlsx Characteristics of the bacterial strains used in this work.

Table S2 aac.01207-25-s0003.xlsx Statistics of MEC^R^ colony frequencies of Kp1, its derivative strains, and *E. coli* MG1655.

Table S3 aac.01207-25-s0004.xlsx Statistics of MEC^R^ colony frequency of Kp1 and MG1655 expressing blaSHV-11 on the monocopy plasmid paGIBAC1.

Table S5 aac.01207-25-s0005.xlsx MEC^R^ mutated strains.

Table S7 aac.01207-25-s0006.xlsx Kp1 genes in the 802-kb chromosomal duplication.

Table S10 aac.01207-25-s0007.xlsx Quantitative parameters of MEC^R^ mutant growth.

ASM does not own the copyrights to Supplemental Material that may be linked to, or accessed through, an article. The authors have granted ASM a non-exclusive, world-wide license to publish the Supplemental Material files. Please contact the corresponding author directly for reuse.
