# Supplementary material for: The mecillinam resistome in Klebsiella pneumoniae: how resistance to a one-target β-lactam triggers a diversity of responses
Source: Antimicrob Agents Chemother. 2026 Feb 3;70(3):e01207-25. doi: 10.1128/aac.01207-25 (PMC12959161; doi:10.1128/aac.01207-25)
Supplement: Supplemental Material — Additional supplemental tables and Fig. S1 to S7. [file aac.01207-25-s0001.pdf]

The mecillinam resistome in *Klebsiella pneumoniae*: how resistance to a one-target β-lactam triggers a diversity of responses.

Marie ROYER[a,b#], Nicolas CABANEL[a], Arnaud GUTIEREZ[c], Guilhem ROYER[a*], Olivier BARRAUD[d], Thierry NAAS[e], Isabelle ROSINSKI-CHUPIN[a], Claude LOVERDO[f,g], Philippe GLASER[a#]

SUPPLEMENTAL MATERIAL

Table S1. Characteristics of the bacterial strains used in this work
Excel file: Table_S1
[&]measured with E-test after 16h of incubation at 37°C. Values are the median of at least three replicates. [#]corresponds to the intersection between the thick and thin layers of bacteria (see Fig 3) ; [$]Mann-Whitney test p-value of the comparison with Kp1(plain) or with Kp1_paGIBAC1 (underlined). * : $p < 0.05$, ns : non-significant.

Table S2. Statistics of MEC^R colony frequencies of Kp1, its derivative strains and *E. coli* MG1655
Excel file: Table_S2
For Fig1 we calculated **(A)** the mean, the standard deviation and the standard error of the MEC^R colony frequencies. Then a three-way ANOVA (**(B)**concentration, **(C)** strain, **(D)** time) was performed and as it was significant we did multiple comparisons tests with Sidak or Tukey methods for p-value adjustments depending on the number of conditions compared. Each subtable (**A, B, C, D**) is shown in one tab.

Table S3. Statistics of MEC^R colony frequencies of Kp1 and *E. coli* MG1655 expressing *bla*$_{SHV-11}$ on the monocopy plasmid paGIBAC1
Excel file: Table_S3
For Fig2 we calculated **(A)** the mean, the standard deviation and the standard error of the MEC^R colony frequencies. Then a three-way ANOVA (**(B)**concentration, **(C)** strain, **(D)** time) was performed and as it was significant we did multiple comparisons tests with Sidak or Tukey methods for p-value adjustments depending on the number of conditions compared. Each subtable (**A, B, C, D**) is shown in one tab.

Table S4. MEC^R colonies appearance rate of clinical *K. pneumoniae* and *E. coli* strains

Strain	#Frequency MEC8	§Frequency MEC64
KP-LIM-005	#3.42 10^{-5} (SD=6.02E-06)	3.53 10^{-5} (SD=8.86E-06)
KP-LIM-006	#9.82 10^{-5} (SD=1.29E-05)	9.40 10^{-5} (SD=5.81E-06)
KP-LIM-008	#1.68 10^{-5} (SD=2.03E-06)	1.46 10^{-5} (SD=3.01E-06)
KP-LIM-012	#3.35 10^{-5} (SD=3.92E-06)	3.23 10^{-5} (SD=4.42E-06)
KP-LIM-014	#8.67 10^{-5} (SD=3.35E-05)	7.83 10^{-5} (SD=3.03E-05)
KP-LIM-062	#6.04 10^{-5} (SD=1.54E-05)	6.60 10^{-5} (SD=1.96E-05)
KP-LIM-146	#3.74 10^{-5} (SD=9.68E-06)	3.49 10^{-5} (SD=7.50E-06)
Kp1	#4.48 10^{-5} (SD=7.99E-06)	3.89 10^{-5} (SD=7.65E-06)
Ec MG1655	&1.35 10^{-5} (SD=5.45E-06)	-
Ec ARDIG-75	&3.42 10^{-6} (SD=1.22E-06)	-

#After 18h of growth on MH plates for the 8 *K. pneumoniae* strains and at 44h of growth for the two *E. coli* strains; § after 18h of growth for the *K. pneumoniae* strains, no colony were detected even after 44h for the two *E. coli* strains. Standard deviation is in parenthesis.

Table S5. MEC^R mutated strains

Excel file: Table_S5

*Position of the nucleotide mutation; $Amino acid position and changes are indicated for single nucleotides polymorphism, indels and intergenic mutation as nucleotide code; &MEC concentration at which mutants were isolated. Mutants highlighted in green have been grown in the presence of MEC (40 µg/mL) during the storage process. In bold, the six mutants selected for in-depth analysis.

Table S6. Distribution of mutations in the second pool of 14 Kp1 MEC^R mutants with MEC pressure kept during storage process.

Category	Gene	Function	Nbr*	Mutations[&]	Nb[#]
Cell wall	*tolQ*	Inner membrane protein, Tol/Pal system	2	Q70*	1
				Y84*	1
Amino acids metabolism	*glnD*	Bifunctional uridylyltransferase/uridylyl-removing	1	G848V	1
Translation	*rRNA 23S*	/	1	G→A non coding	1
	spoT	Bifunctional (p)ppGpp synthetase and hydrolase	1	Q367P	1
	aspS	aspartate tRNA ligase	1	T3S	1
	tRNA Ser	/	1	C→A	1
Intergenic mutations	*ENJAKFCB_02626 / ENJAKFCB_02627*	Hypothetical proteins	1	C→A	1
	dauA/prs	dauA : C4-dicarboxylic acid transporter ; prs : ribose-phosphate pyrophosphokinase	1	C→A	1
Amplifications	*ENJAKFCB_03869 to ENJAKFCB_03932*	64 phage genes (S. Table 4)	2	43 kb	2
	Large duplication near ribosomal operon	720 genes (S. Table 4.5)	6	802 kb	6

[&] One letter amino acid code, stars represent a stop codon; *Number of isolates mutated in the gene.
[#]Number of isolates showing the same mutation.

Table S7. Kp1 genes in the 802 kb chromosomal duplication.
Excel file: Table_S7

Table S8. Distribution of mutation in 37 sequenced MEC^R mutants from Kp1Δ*bla*$_{SHV}$.

Category	Gene	Function	Nbr*	Mutations	Nb$	Kp 1&
Cell wall (14%#)	*cpxA*	Sensor protein, Cpx system	1	Q153K	1	17
	tolA	Inner membrane protein, Tol/Pal system	1	Q425*	1	3
	tolQ	Inner membrane protein, Tol/Pal system	1	E68*	1	0
	igaA	Intracellular growth attenuator protein, RCS system	1	Δ15 bp. nt 144	1	0
	mreB	Rod system	1	(AGCGCGT)$_{1\rightarrow2}$. nt 981	1	0
Amino acids metabolism (27%)	*cysB*	Transcriptional regulator, L-cystein biosynthesis	8	IS6. nt 108/251/412	7	6
				128 bp inversion nt 230	1	
	cysE	Serine acetyl-stranferase, L-cystein biosynthesis	1	Δ54 bp nt 615	1	1
	ntrB (glnL)	Sensor, nitrogen regulation	1	Q119*	1	3
Central carbon metabolism (8%)	*lipA*	lipoyl synthase	1	V103F	1	2
	lpdA	Pyruvate dehydrogenase subunit E3	1	(TTGGTGCCGACAATCG CGCCGCCG)$_{1\rightarrow2}$ nt 1227	1	0
	cyaA	adenylate cyclase	1	Δ1 bp nt 2246	1	0
Translation (22%)	*epmB*	L-lysine 2,3 amino-mutase	1	IS903 nt 891	1	1
	lysS	Lysine tRNA ligase	3	A283V	3	0
	tRNA Ser	/	2	G→A nt 67	1	0
				C→A nt 62	1	
	argS	arginyl tRNA ligase	1	A457T	1	0
	aspS	aspartate tRNA ligase	1	T162A	1	0
	rpoB	RNA polymerase ß subunit	1	T553P	1	0
	rpoN	Sigma factor	1	G152D	1	0
Intergenic mutations (5%)	yecD / *aspS*	yecD : isochorismatase family protein ; aspS : aspartate tRNA ligase	1	2 bp→AA (aspS promotor) nt -160	1	0
	mltB / ENJAKFCB_01056	murein lytic transglycosylase	1	C→T intergenic nt 230	1	0
Others (35%)	No mutation	/	1	/		25
	Large duplication near ribosomal operon	720 genes (see supp 4.5)	7	802 kb	7	6
	ENJAKFCB_03869 to ENJAKFCB_03932	64 phage proteins (see supp 4)	2	43 kb	2	2
	codB	Cytosine permease	2	T307P	2	0
	cydD	Cystein/glutathione ABC transporter permease	1	S405P	1	1

#Percentages are calculated with the total number of sequenced mutants, as some mutants harbored several mutations, the sum of the percentages is different from 100%. *Number of isolates mutated in the gene. $Number of isolates with the same mutation in the same gene. &Number of isolates mutated in the same gene in the Kp1 MEC^R collection (Table 1)

Table S9. Impact of *bla*$_{SHV-11}$ on MEC MIC

Strain	MEC#	MEC+Clav*
Kp1	0.1095 (0.02)	0.1095 (0.02)
Kp1Δ*bla*$_{SHV-11}$	0.1095 (0.02)	0.1095 (0.02)
aceF	>256	8 (0)
cpxA	>256	48 (0)
ntrC	>256	10 (2.83)
tolB	>256	12 (0)
cysB	>256	48 (22.63)
spoT	>256	10 (2.83)

Median value of duplicates, standard deviation in parentheses.
*MH agar plates were supplemented with potassium clavulanate 2µg/mL to measure the MIC by Etest.

Table S10. Quantitative parameters of Kp1, *aceF*, *cpxA*, *ntrC*, *tolB*, *cysB* and *spoT* MEC^R mutants growth in MH broth at 0 to 128 µg/mL MEC.

Excel file: Table_S10
*Two-way ANOVA (Concentration, Strain). MEC compared with MH condition for each mutant. And MH condition for each mutant compared with the MH Kp1 condition. sd = standard deviation; se = standard error.

Table S11. *cpxA* mutations in clinical isolates.

Accession	Description	NCBI entry title	Publication
OW959925.1	Kp isolate 11 genome assembly	No	No publication
CP050336.1	Kp strain ZRKP04	*In Vivo* Selection of Meropenem Resistance among Ceftazidime-Avibactam-Resistant Meropenem- Susceptible *Klebsiella pneumoniae* Isolate with KPC-33 Carbapenemase	Wang *et al.,* 2021(1)
CP049719.1	Kp strain 06KM907	Whole genome sequencing of global problem clones of OXA-48-producing *Klebsiella pneumoniae* from the veterinary setting in Switzerland	Brilhante *al,*. 2021(2)
CP103687.1	Kp strain ST307	Systematic Analysis of Mobile Genetic Elements Mediating Beta-lactamase Gene Amplification in Non-Carbapenemase-Producing Carbapenem Resistant Enterobacterales Bloodstream Infections	Shropshire *et al.,* 2022(3)
CP128725.1	Kp strain CRE-243	*Klebsiella pneumoniae* Clinical Isolates with Features of Both Multidrug-Resistance and Hypervirulence Have Unexpectedly Low Virulence	Kochan *et al.*, 2023(4)
CP060744.1	Kp strain KP120	*In Vivo* Evolution of CTX-M-215, a Novel Narrow-Spectrum-Lactamase in an *Escherichia coli* Clinical Isolate Conferring Resistance to Mecillinam	Yin *et al.*, 2020(5)
CP106786.1	Kp strain KP1161	Genomic definition of *Klebsiella pneumoniae* KP1161	No publication
CP089765.1	Kp strain HKC2	Establishing three clusters in ESBL and KPC-producing hypervirulent/ hypermucicious *Klebsiella pneumoniae* isolates in China	No publication
CP071385.1	Kp strain wxc2020	*Klebsiella pneumoniae* AmpC producer resistant to ceftazidime-avibactam	No publication
CP069821.1	Kp strain FDAARGOS_1326	FDA dAtabase for Regulatory Grade micrObial Sequences (FDA-ARGOS): Supporting development and validation of Infectious Disease Dx tests	No publication
CP128770.1	Kp strain CRE-017	*Klebsiella pneumoniae* Clinical Isolates with Features of Both Multidrug-Resistance and Hypervirulence Have Unexpectedly Low Virulence	Kochan *et al.*, 2023(4)
CP079599.1	Kp strain WRC10_CMC309MC	Complete genome of Carbapenem resistant *Klebsiella pneumoniae*_WRC10_CMC309MC	No publication
CP166329.1	Kp strain CRE378	*Klebsiella pneumoniae* strain: CRE378 Genome sequencing and assembly. Carbapenem Resistant Hypervirulent Klebsiella pneumoniae	No publication
CP139932.1	Kp strain HZKP1	Genomic and phylogenetic analysis of a NDM-1 producing ST152 *Klebsiella pneumoniae* isolated from a bloodstream infection in China	Fang *et al.*, 2025(6)
CP079599.1	Kp stain A28441	Epidemiology of chromosomes and plasmids of multidrug-resistant bacteria	Sobkowiak *et al.*, 2025(7)

Table S12. Plasmids.

Plasmid	Characteristics	Origin
p15Red	p15A ori, *araBAD*, CmR, λred recombinase	Lab collection
pAGIBAC1	Derivative from pINDIGOBAC-536, IncFIA, *oriS*, *sopABC*, cos, *lacZ*, CmR	Centre National de Ressources Génomiques Végétales, INRAE Occitanie-Toulouse, Arnaud Bellec
IRC178	*ompF*, kanaR, FRT	Lab collection
MiniTn-Zeo	oriR6K, *araBAD*, ZeoR, FRT	Lab collection
pAGIBAC1_empty	IncFIA, *oriS*, *sopABC*, cos, CmR	This work
pAGIBAC1_$bla_{\text{SHV-11}}$	IncFIA, *oriS*, *sopABC*, cos, CmR, $bla_{\text{SHV-11}}$ under its own promotor	This work

Table S13. Primers.

Name	Sequence (5'-3')	Target
MR25	aaggagaggacaatggttgcggtaagaagtgcacatcttaataaagctggtcag gcatgcttcgaagttc	kanaR_relA F
MR26	ttatccggcctgcagatgaattaaccgccgtgcagacggcgcgcgtcgatggag tggtgaatccgttagc	kanaR_relA R
MR19	tcatcaaacttgctgagcgt	relA F
MR20	tttcgatctgatcgcccatc	relA R
MR35	tgatgtaccgctaatgccccttttgagagatgaaattcgcgaccactccgtcag gcatgcttcgaagttc	kanaR_mrdA_2 F
MR36	tgcggaataagtcctgtcgcttactgggcatcggcgctatcacgctgcggggag tggtgaatccgttagc	kanaR_mrdA_2 F
MR37	aacctttcagtagggctgac	mrdA_2 F
MR38	cggttacgtgtcacctttc	mrdA_2 R
MR55	cactcaaggatgtattgtggttatgcgttatattcgcctgtgtattatctaatt ggggatcttgaagtac	zeoR_bla$_{SHV}$ F
MR56	cggccaccgccgggttagcgttgccagtgctcgatcagcgccgcgccgatgtga agaaaaagtgaatgat	zeoR_bla$_{SHV}$ R
MR45	tcgcttctttactcgccttt	bla$_{SHV}$ F
MR46	tcaccaccatcattaccgac	bla$_{SHV}$ R
MR99	ccgcgtaggcatgatagaaa	bla$_{SHV}$ intra F
MR100	ccggtcttatcggcgataaa	bla$_{SHV}$ intra R
MR95	gtcgaccaattctcatgtttgacagcttatcatcg	Gibson_vector F
MR96	gccccgacacccgccaac	Gibson_vector R
MR97	Ggcgggtgtcggggcattattcgaaataaaagatgacaaatgatgaaggaaaaa agaggaa ttgtgaatcagcaaaac	Gibson_bla$_{SHV}$ F
MR98	catgagaattggtcgaccgcggccaccgccgggtt	Gibson_bla$_{SHV}$ R
MR103	taactgcgcgcggtcgaccaattctcat	Reverse_vector F
MR104	cgcagttattcgaataatgccccgacac	Reverse_vector R
MR105	ttttagagcgccttgtaggc	paGIBAC F
MR106	tctcgcagataccgtcatgt	paGIBAC R
IRC675	cagtacatcagcgatcacct	p15Red (araC) F
IRC676	gtcttcgtgcctgtcaaatg	p15Red (araC) R
IRC1153	aggataactatcagctctatctgattcgtcccgccagcacctcacaatccgact ttattaacatgggaattagccatggtc	Kmtse_cpxA_F
IRC1154	tcgacgttaaccgtaatcccgtctttatccaccgagaagctgacggaaattttc gtatgctggagctgcttcgaag	Kmtse_cpxA_R
IRC1155	gataacgccgatcatccgca	cpxAm_F
IRC1156	ggaaaatctgttcgcgatcct	cpxAm_R
ICR1157	gttctaaactggcctacgttacctttgaaagcggtcgttctgcgctggtggttc agacatgggaattagccatggtc	Kmtse_tolB_F
IRC1158	gacttggcgtttcgtctaaaaacgttgatgacagaacctgtacaccacccgttt ccagatcgctggagctgcttcgaag	Kmtse_tolB_R
IRC1159	ccagttcctggttcaccgt	tolBm_F
IRC1160	tccatcagttgccggaagac	tolBm_R
IRC1161	aaactacaacagcttcgctacatcgttgaggtggttaaccataacctgaatgtt tcatccacatgggaattagccatggtc	Kmtse_cysB_F
IRC1162	gcaaaacgttgaataaaatcatacatgtagcttcgtaagaaggtgctacggcgg aaacctagctggagctgcttcgaag	Kmtse_cysB_R
IRC1163	cgacaagttgcctgaacgt	cysBm_F
IRC1164	atctaccacgtccctggt	cysBm_R
IRC1169	cgtttgattatttgcccaaaccttttgatatcgatgaagccgtcgccctcgtcg acatgggaattagccatggtc	Kmtse_ntrC_F
IRC1170	ggggacgatcatactcaactcatcggaacagtaaagtacaaaatagcagcactt tgctggagctgcttcgaag	Kmtse_ntrC_R
IRC1171	atccaatgcttccggtcat	ntrCm_F
IRC1172	gatgcagcagttctcacg	ntrCm_R

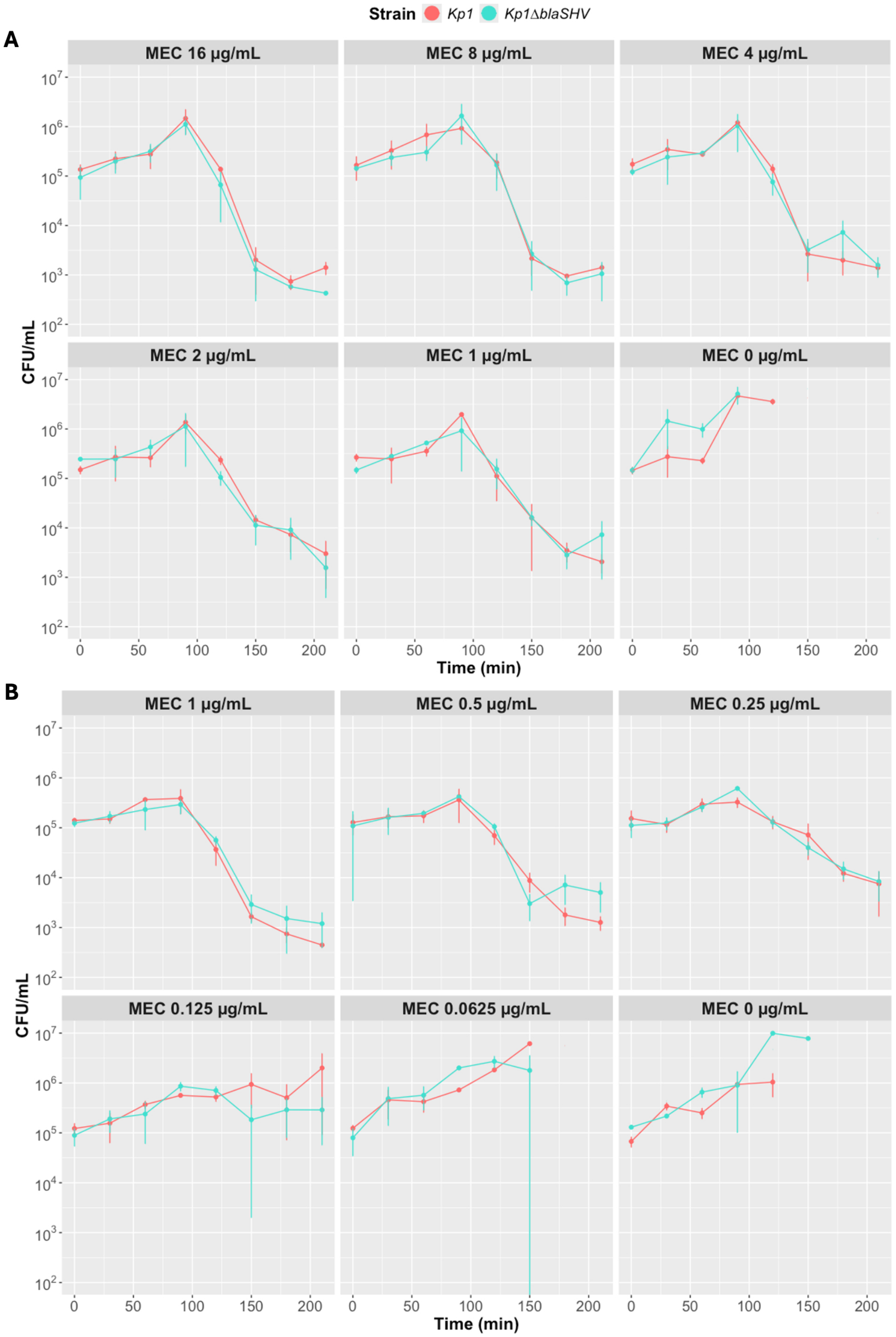

Figure S1. MEC time-kill experiment of Kp1 and Kp1Δ*bla*$_{\text{SHV-11}}$. (A) MEC concentrations ranging from 1 µg/mL to 16 µg/mL and (B) concentrations ranging and from 0.0625 µg/mL to 1 µg/mL. The two strains Kp1 (red) and KP1Δ*bla*$_{\text{SHV-11}}$ (blue) showed similar MEC MIC of 0.22 and 0.19 mg/L respectively (S1 Table). Each point represents the average value and the error bars the standard error of two independent experiments.

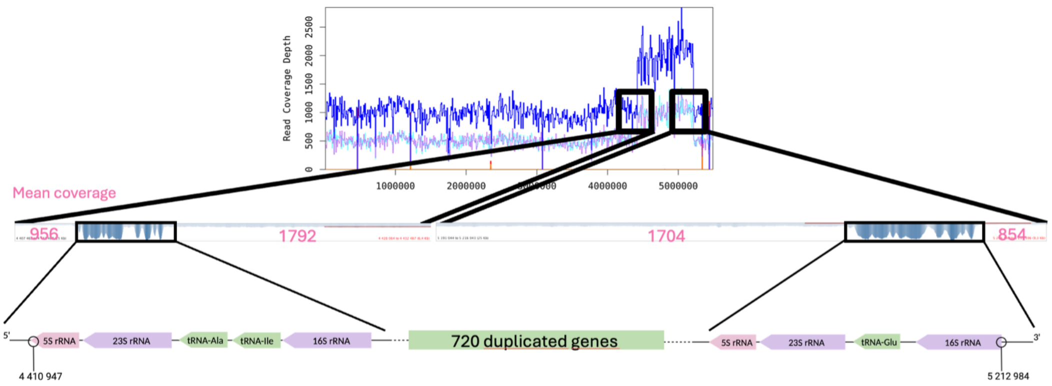

Figure S2. Detection based on read coverage of a large DNA amplification between two ribosomal operons. The upper panel represents the read coverage depth (in dark blue) along Kp1 chromosome as calculated by using Breseq (8). In the lower panel, zoom on the amplified region showing on both ends ribosomal operons. Numbers in black in each panel represent the distance in nucleotide from the predicted origin of replication. Numbers in pink (lower panel) indicate the mean coverage depth of reads calculated with Tablet (9).

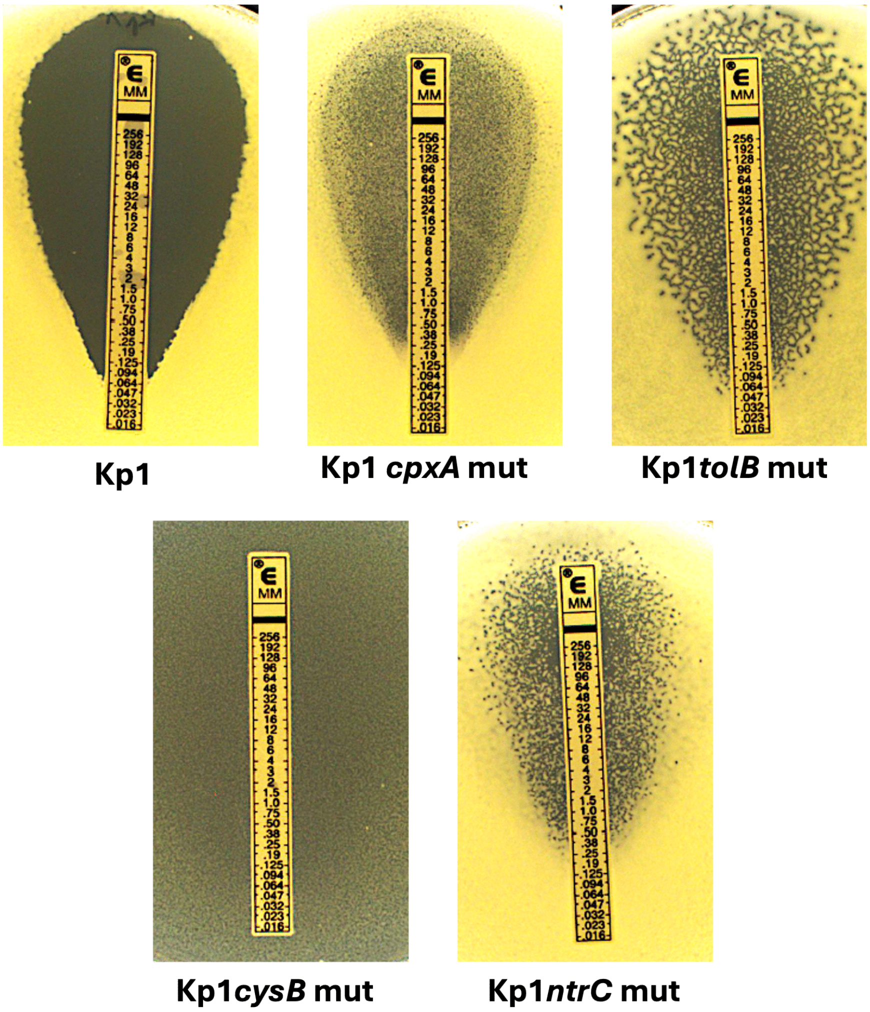

Figure S3. MEC MIC of Kp1 and reconstructed *cpxA*, *tolB*, *cysB* and *ntrC* mutants. MIC (µg/mL) were measured by Etest® after 16h of incubation at 37°C with the flooding method. Experiments were performed in duplicates. Median MIC values are given in S1 Table.

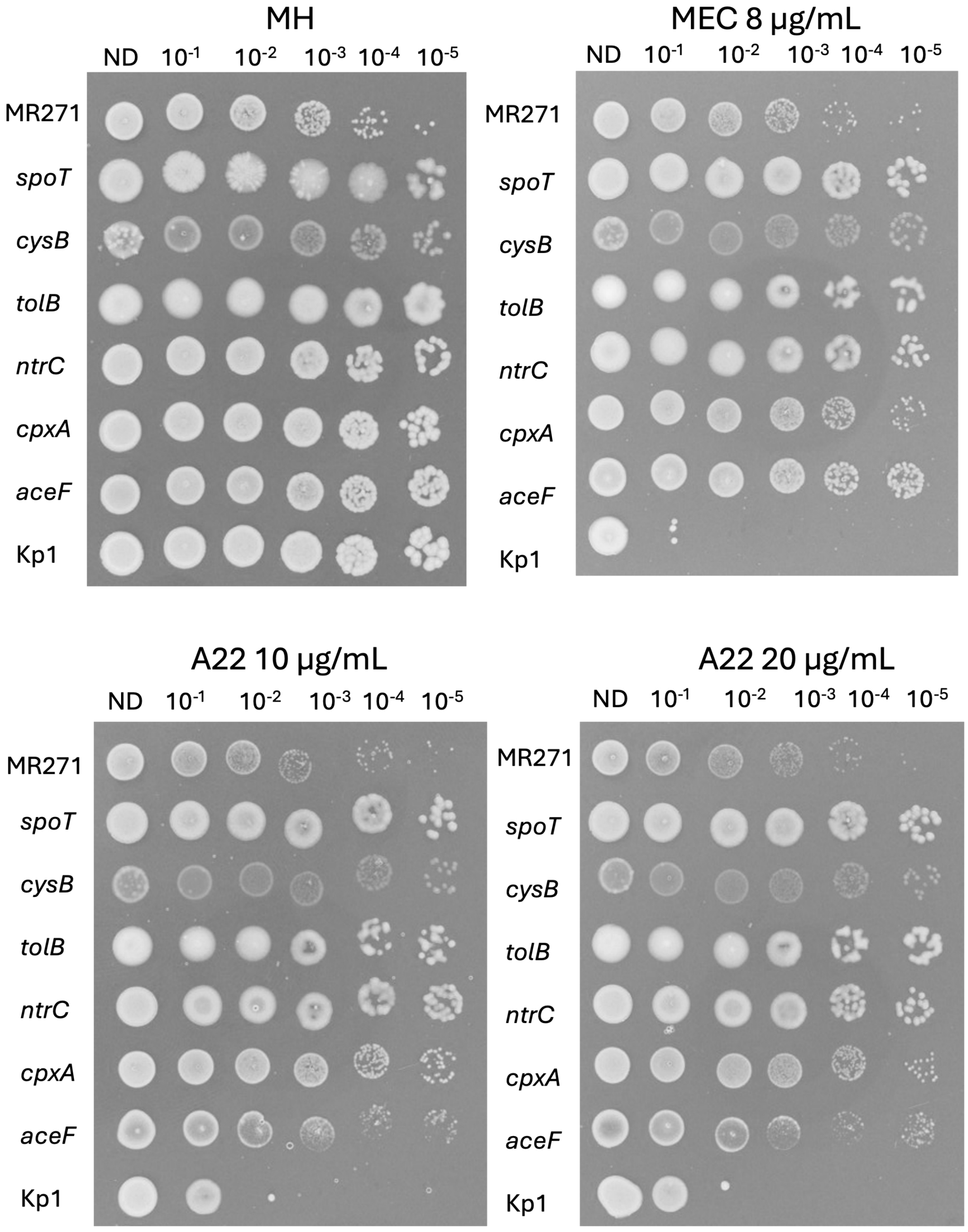

Figure S4. A22 and mecillinam susceptibility of Kp1 and MEC^R mutant derivatives. The eight strains were grown until stationary phase and diluted as indicated above each image. The non diluted suspension was normalized at an OD_{600} of 0.2. Four µl were spotted on MH agar, supplemented or not with MEC (8 µg/mL) or the MreB inhibitor A22 (10 or 20 µg/mL). Plates were incubated at 37 °C for 19 hours. The growth inhibition observed is representative of three independent experiments. MR271 strain harbors the 720 genes duplication.

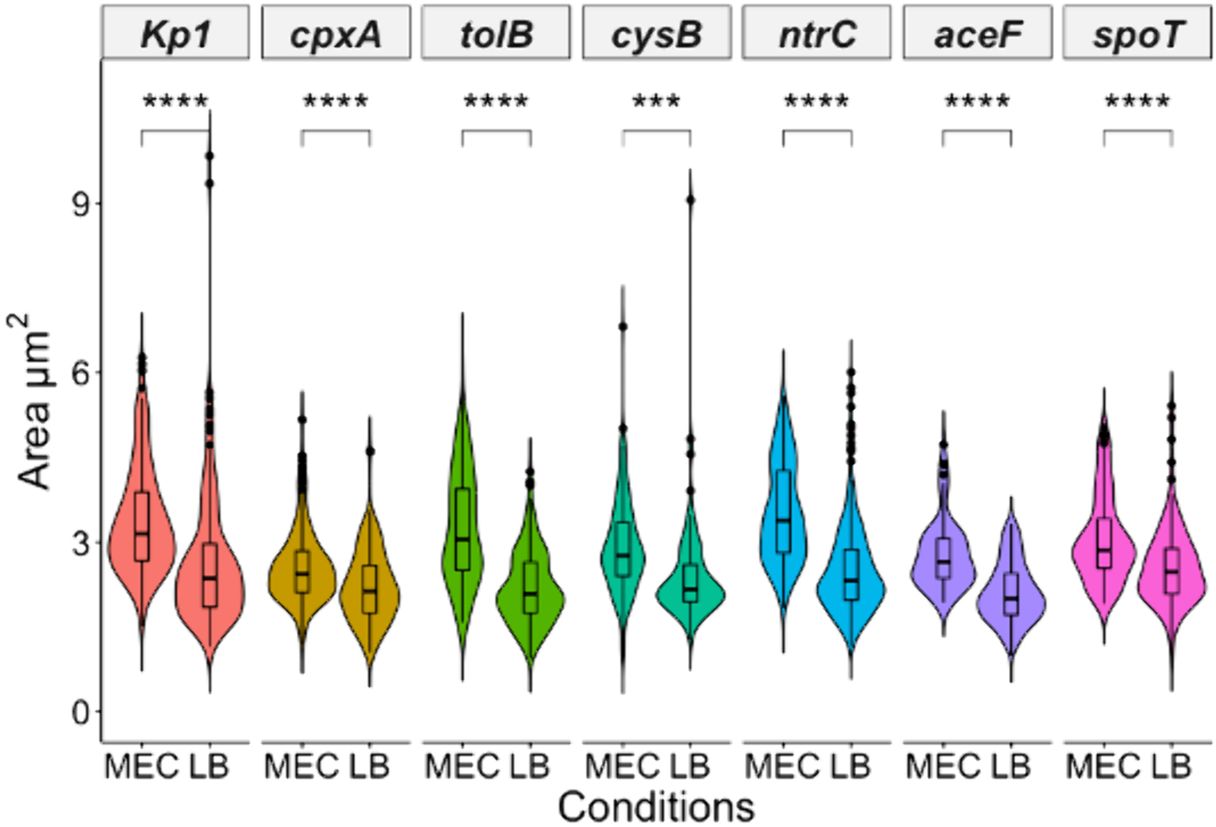

Figure S5. The impact of MEC on the area of Kp1, *aceF*, *cpxA*, *ntrC*, *tolB*, *cysB* and *spoT* mutants cells. Three-hour time-lapse microscopy experiments were performed on LB agarose pads supplemented or not with MEC 10µg/mL. Phase contrast observations were done on a Nikon inverted Tie microscope with images taken every five minutes with a sCMOS ORCA flash 4.0 camera. Violin plot of the distributions of bacterium area at 40 min on LB and on LB+MEC pads with boxplots representing from top to bottom the 3rd quartile, the median and the 1st quartile on eight different stacks. Black dots represent predicted outliers. Picture analysis was done in Fiji with the MicrobeJ plug-in (10). A Student test was performed to compare the area for each strain on LB+MEC with LB. *** $p < 0.005$, ****$p < 0.001$.

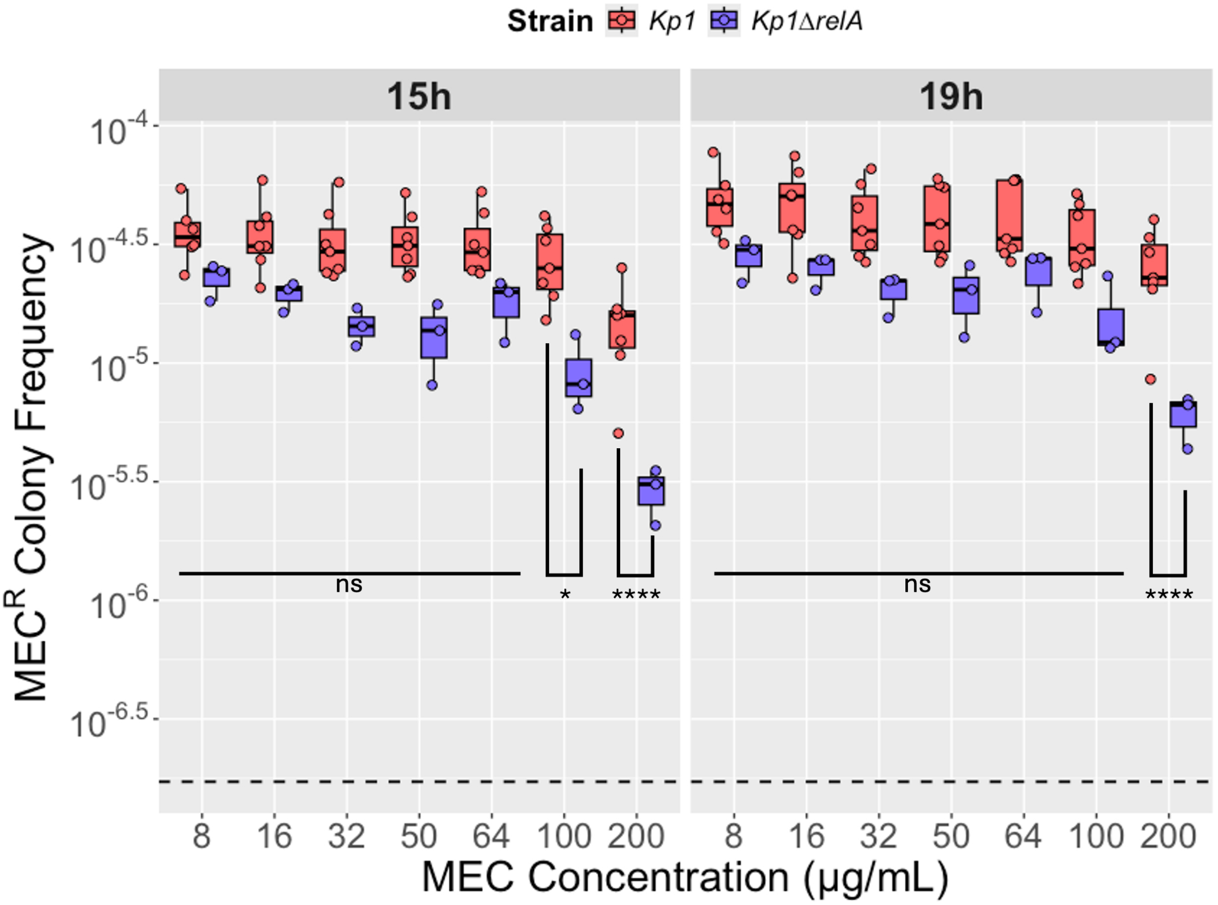

Figure S6. MEC^R colony frequency of Kp1 and Kp1Δ*relA*. MEC^R colony frequency of Kp1 (red) and Kp1Δ*relA* (purple). Diluted bacterial cultures were plated on MH agar plates supplemented with MEC at 8/16/32/50/64 and 100 and 200 mg/L overnight at 37°C. CFUs were counted at two time points: 15h and 19h30 post-incubation. The MEC^R colony frequency for one strain is the ratio between CFUs/mL on MH-MEC and CFUs/mL on MH. Experiments were performed with at least three replicates. Boxplots are representing from top to bottom the 3rd quartile, the median and the 1st quartile. The dash line corresponds to the lowest MECR colony frequency that can be measured (corresponding to one colony growing on MH+MEC plate). A three-way ANOVA (strain, time, concentration) was performed and as it was significant we did multiple analysis with Sidak or Tukey methods for p-value adjustments depending on the number of conditions compared. Here, we only show the comparison of the two strains at each MEC concentrations and time points: *** $p < 0.005$, **** $p < 0.001$, ns=non-significant.

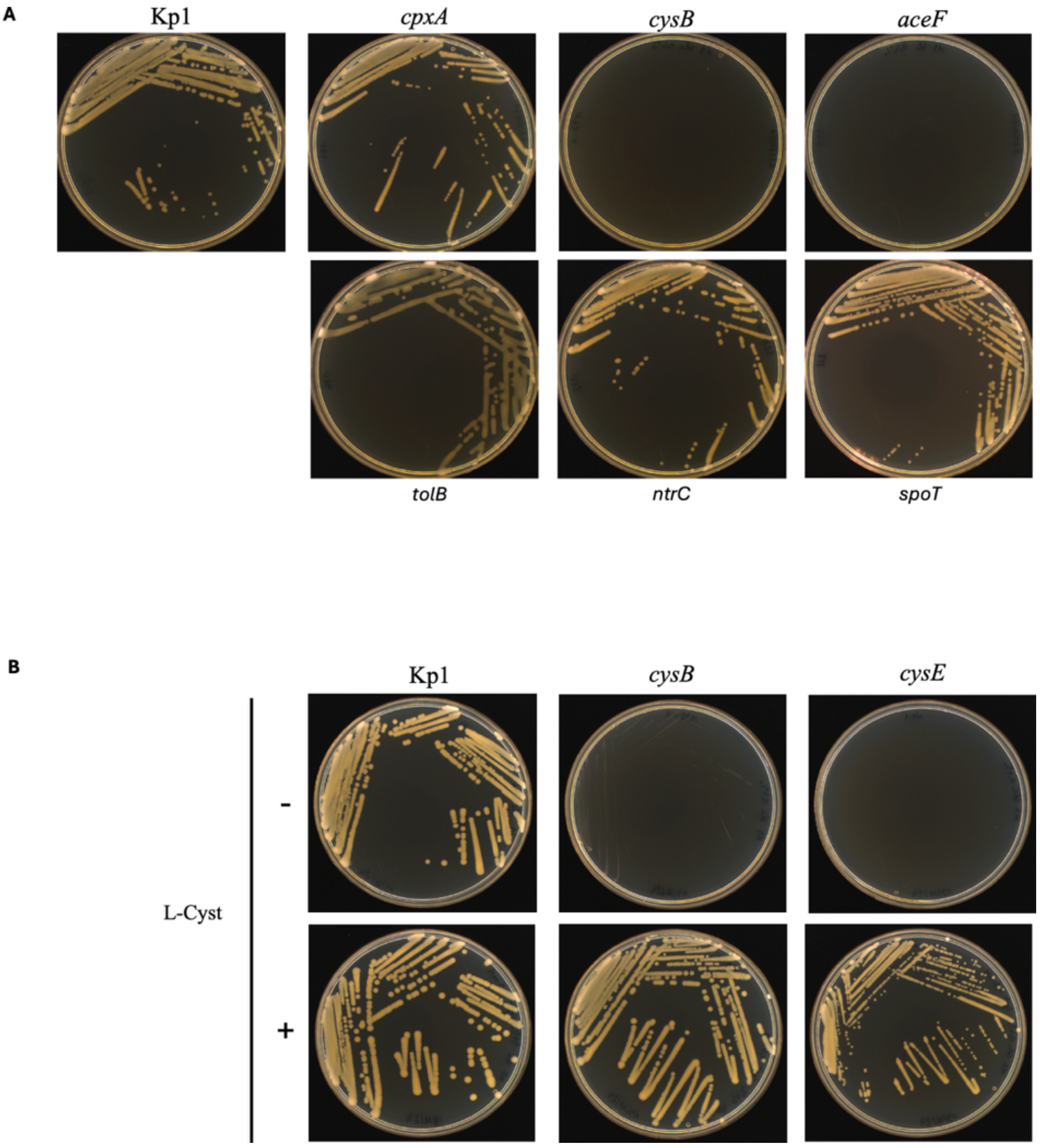

Figure S7. Mutant strain auxotrophy screen. (A) Strains were streaked on M9 agar plates supplemented with glucose 0.4%. (B) To confirm the L-cysteine auxotrophy of the *cysB* and *cysE* mutants, strains were streaked in parallel on glucose 0.4% M9 agar supplemented or not with 0.5 mM L-cysteine. Plates were incubated at 37°C for 24h. The lack of growth of the *aceF* mutant, which is deficient in pyruvate dehydrogenase, is due to a defect in carbon metabolism. Experiments have been done in two replicates.

References

1. Wang C, Zhao J, Liu Z, Sun A, Sun L, Li B, Lu B, Liu Y, Cao B. 2021. In vivo Selection of Imipenem Resistance Among Ceftazidime-Avibactam-Resistant, Imipenem-Susceptible *Klebsiella pneumoniae* Isolate With KPC-33 Carbapenemase. Front Microbiol 12:727946.

2. Brilhante M, Gobeli Brawand S, Endimiani A, Rohrbach H, Kittl S, Willi B, Schuller S, Perreten V. 2021. Two high-risk clones of carbapenemase-producing *Klebsiella pneumoniae* that cause infections in pets and are present in the environment of a veterinary referral hospital. J Antimicrob Chemother 76:1140–1149.

3. Shropshire WC, Konovalova A, McDaneld P, Gohel M, Strope B, Sahasrabhojane P, Tran CN, Greenberg D, Kim J, Zhan X, Aitken S, Bhatti M, Savidge TC, Treangen TJ, Hanson BM, Arias CA, Shelburne SA. 2022. Systematic Analysis of Mobile Genetic Elements Mediating β-Lactamase Gene Amplification in Noncarbapenemase-Producing Carbapenem-Resistant Enterobacterales Bloodstream Infections. mSystems 7:e00476-22.

4. Kochan TJ, Nozick SH, Valdes A, Mitra SD, Cheung BH, Lebrun-Corbin M, Medernach RL, Vessely MB, Mills JO, Axline CMR, Nelson JA, VanGosen EM, Ward TJ, Ozer EA, van Duin D, Chen L, Kreiswirth BN, Long SW, Musser JM, Bulman ZP, Wunderink RG, Hauser AR. 2023. *Klebsiella pneumoniae* clinical isolates with features of both multidrug-resistance and hypervirulence have unexpectedly low virulence. Nat Commun 14:7962.

5. Yin M, Hu G, Shen Z, Fang C, Zhang X, Li D, Doi Y, Zhang Y, Wang M, Guo Q. 2020. In Vivo Evolution of CTX-M-215, a Novel Narrow-Spectrum β-Lactamase in an *Escherichia coli* Clinical Isolate Conferring Resistance to Mecillinam. Antimicrob Agents Chemother 64:10.1128/aac.00562-20.

6. Fang R, Di L, Tian X, Bai Y. 2025. Genomic and phylogenetic analysis of a NDM-1 producing ST152 *Klebsiella pneumoniae* isolated from a bloodstream infection in China. J Glob Antimicrob Resist 40:34–36.

7. Sobkowiak A, Schwierzeck V, van Almsick V, Scherff N, Schuler F, Bessonov K, Robertson J, Harmsen D, Mellmann A. 2025. The dark matter of bacterial genomic surveillance—antimicrobial resistance plasmid transmissions in the hospital setting. J Clin Microbiol 63:e00121-25.

8. Deatherage DE, Barrick JE. 2014. Identification of Mutations in Laboratory-Evolved Microbes from Next-Generation Sequencing Data Using breseq, p. 165–188. *In* Sun, L, Shou, W (eds.), Engineering and Analyzing Multicellular Systems: Methods and Protocols. Springer, New York, NY.

9. Milne I, Bayer M, Stephen G, Cardle L, Marshall D. 2016. Tablet: Visualizing Next-Generation Sequence Assemblies and Mappings, p. 253–268. *In* Edwards, D (ed.), Plant Bioinformatics: Methods and Protocols. Springer, New York, NY.

10. Ducret A, Quardokus EM, Brun YV. 2016. MicrobeJ, a tool for high throughput bacterial cell detection and quantitative analysis. Nat Microbiol 1:16077.
